# An in silico approach to elucidate the pathways leading to primary osteoporosis: age-related vs. postmenopausal

**DOI:** 10.1007/s10237-024-01846-2

**Published:** 2024-05-03

**Authors:** Rocío Ruiz-Lozano, José Luis Calvo-Gallego, Peter Pivonka, Michelle M. McDonald, Javier Martínez-Reina

**Affiliations:** 1 Departmento de Ingeniería Mecánica y Fabricación, Universidad de Sevilla, 41092 Seville, Spain; 2 School of Mechanical, Medical and Process Engineering, Queensland University of Technology, Brisbane, QLD 4000 Australia; 3 Faculty of Medicine and Health, The University of Sydney, Sydney, NSW Australia

**Keywords:** Age-related osteoporosis, Postmenopausal osteoporosis, RANKL-RANK-OPG signalling pathway, Oestrogen deficiency, TGF-$\upbeta$

## Abstract

Numerical models of bone remodelling have traditionally been used to perform in silico tests of bone loss in postmenopausal women and also to simulate the response to different drug treatments. These models simulate the menopausal oestrogen decline by altering certain signalling pathways. However, they do not consider the simultaneous effect that ageing can have on cell function and bone remodelling, and thus on bone loss. Considering ageing and oestrogen decline together is important for designing osteoporosis treatments that can selectively counteract one or the other disease mechanism. A previously developed bone cell population model was adapted to consider the effect of ageing through: (1) the decrease of TGF-$\upbeta$ contained in the bone matrix and (2) an increased production of sclerostin by non-skeletal cells. Oestrogen deficiency is simulated in three different ways: (a) an increase in RANKL expression, (b) a decrease in OPG production, and (c) an increase in the responsiveness of osteoclasts to RANKL. The effect of ageing was validated using the cross-sectional study of (Riggs et al. in J Bone Miner Res 19: 1945-1954, 2004) on BMD of trabecular bone of the vertebral body of men. The joint effect of ageing and oestrogen deficiency was validated using these same clinical results but in women. In ageing, the effect of the increasing production of sclerostin is more important than the decrease of TGF-$\upbeta$, while the three mechanisms used to simulate the effect of oestrogen deficiency produce almost identical responses. The results show that an early menopause leads to a lower average density in the fifth decade, but after the sixth decade the average density is independent of the age at menopause. Treatment of osteoporosis with denosumab was also simulated to conclude that the drug is not very effective if started before 10 years after menopause or before age 60.

**Supplementary Information:**

The online version contains supplementary material available at 10.1007/s10237-024-01846-2.

## Introduction

Osteoporosis is a disease caused by an imbalance in the remodelling process. Resorption of the old bone, carried out by osteoclasts, predominates over bone formation by osteoblasts. This leads to a net bone loss and a decrease in the stiffness and strength of bones, increasing the risk of fracture. Primary osteoporosis is the bone loss that occurs during the normal human ageing process, while secondary osteoporosis is defined as the bone loss that results from certain clinical disorders or treatments (Fitzpatrick 2002).

Primary osteoporosis is the most common form of the disease and includes gonadal insufficiency-related osteoporosis (type I), such as postmenopausal osteoporosis (PMO), and senile osteoporosis (type II) (Dobbs et al. 1999), also called age-related osteoporosis (ARO). PMO is caused by oestrogen deficiency occurring after menopause, which has been reported to alter the bone remodelling process via different mechanisms, namely: 1) through an enhanced RANKL expression by stromal cells and lymphocytes (Eghbali-Fatourechi et al. 2003); 2) through a decrease of the secretion of OPG by osteoblasts, which is enhanced by oestrogen receptor agonists in a normal state (Hofbauer et al. 1999; Viereck et al. 2003) and consequently it must be reduced upon oestrogen deficiency; and 3) since oestrogen suppresses the responsiveness of osteoclasts to RANKL (Shevde et al. 2000), oestrogen deficiency will increase that responsiveness, i.e. osteoclasts will be differentiated at lower RANKL concentrations (Menaa et al. 2000).

In turn, ARO has been attributed or related to several factors: (1) a decreasing concentration of $TGF-\upbeta$ in bone matrix with age without differences between genders (Nicolas et al. 1994); (2) an increasing concentration of serum sclerostin with age which suppresses bone formation (Ardawi et al. 2011); (3) an increased rate of osteocyte apoptosis (Milovanovic and Busse 2020); and (4) cellular senescence (Farr and Khosla 2019).

The factors leading to ARO and PMO are superposed in women. In men, a similar effect to that of menopause is likely to occur, in this case affecting the androgen levels. Androgens upregulate $TGF-\upbeta$ and insulin-like growth factors (IGFs), which stimulate bone formation (Kasperk et al. 1990, 1997), and downregulate interleukin IL-6 (Bellido et al. 1995) and PTH (Pilbeam and Raisz 1990), which stimulate osteoclastogenesis. Thus, the overall effect of androgens is to increase bone mass and their deficiency (andropause) has the opposite effect. However, unlike middle-aged women, middle-aged men do not experience a sudden halt in gonadal function and the decline in androgen levels is continuous over time (Vermeulen 2000). This results in a different pattern of bone loss with age in women and men, as reported by Riggs et al. (2004). In a cross-sectional study involving 373 women and 323 men (age, 20–97 years), these authors measured volumetric bone mineral density (vBMD) by QCT at different anatomic sites. For the vertebral body they observed a steady decline with age in both men and women, but with a more pronounced bone loss in women around menopause.

Nordin et al. separated the effect of age and years since menopause (YSM) by measuring forearm bone density in postmenopausal women aged 33 to 75 years and whose age at menopause could be established. To this end they extrapolated the density decline due to age, suggesting that the influence of age is sustained over time, resulting in an approximately linear decrease in bone density that is superposed to a logarithmic decline with YSM. This would be very pronounced in the first years after menopause but it would slow down in the long term (Nordin et al. 1990).

Several mathematical models have been proposed in the literature to predict the bone loss due to age (Lemaire et al. 2004) and menopause (Scheiner et al. 2014; Martínez-Reina and Pivonka 2019; Martínez-Reina et al. 2021a, b; Calvo-Gallego et al. 2022). These are bone cell population models (BCPM) that can account for the effects of age or menopause and the subsequent oestrogen deficiency through the biochemical factors that control bone cell differentiation, survival, action and interconnection between different cell types. Regarding PMO, Lemaire et al. assumed that oestrogen deficiency produces a decrease of OPG levels (Lemaire et al. 2004). Several authors have modelled oestrogen deficiency through an increase in the expression of RANKL (Scheiner et al. 2014; Martínez-Reina et al. 2021b). As far as we know, no mathematical model has simulated oestrogen deficiency through an increased responsiveness of RANK. It is not clear which of the three factors (RANKL, OPG and RANK responsiveness) is the most appropriate to model oestrogen deficiency or whether a combination of the three leads to better results. In this work, we intend to use our BCPM (Martínez-Reina et al. 2022) to check if a similar response can be obtained by modelling oestrogen deficiency in any of these three ways.

To our knowledge, only Lemaire et al. simulated the effect of age, particularly with a decrease in $TGF-\upbeta$ concentration (Lemaire et al. 2004). Martin et al. considered an increasing sclerostin concentration with age, but they attributed it to menopause and specifically to a clearance rate decreasing with YSM (Martin et al. 2019). In any case, there is evidence of increasing sclerostin levels with age, independent of menopause (Mödder et al. 2011) and this effect should be accounted for in the analysis of ARO.

Understanding the aetiology of OP and, more specifically, discerning which factors are intrinsic to ARO and which are intrinsic to PMO is key to the design of patient-specific treatments, as both can occur in postmenopausal women, but only some can be counteracted by medication. Current treatments for PMO primarily combat the effects of oestrogen deficiency, but the interaction of these effects with those of ageing is not known and it is important that it be taken into account in the design of treatments. Related to this, it is likely that age and YSM should be considered independent variables in the design of patient-specific treatments. The goal of the present work is to shed light on different approaches to simulating osteoporosis with BCPM and to analyse the interaction between age and the time elapsed since menopause in the progression of the disease in women.

## Materials and methods

### Bone cell population model of bone remodelling

**Fig. 1:**
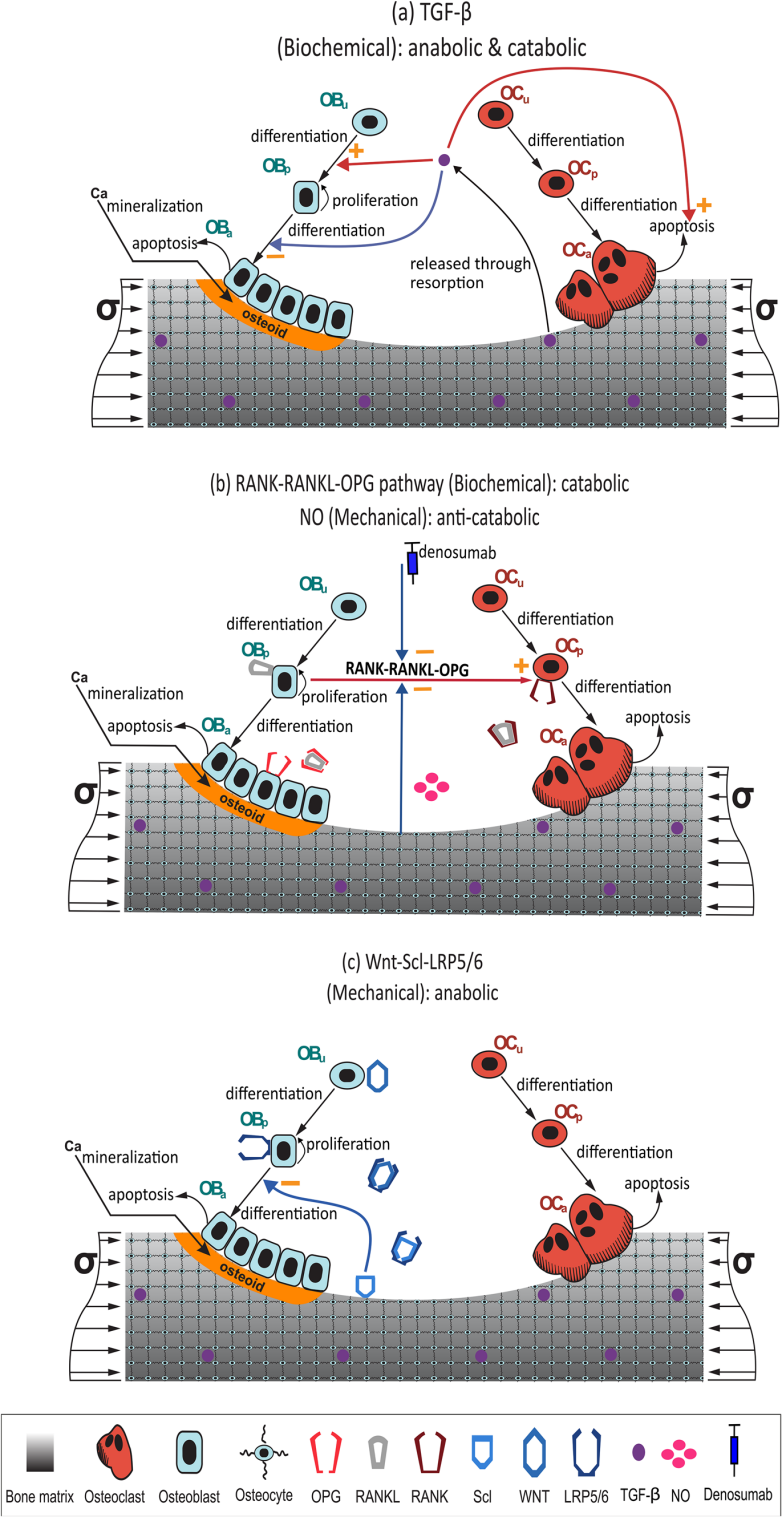
Schematic representation of the bone cell population model: bone cell differentiation stages are presented along with biochemical and biomechanical interactions. The three main mechanisms involved in the model are shown separately: **a** $TGF-\upbeta$, **b** RANK-RANKL-OPG signalling pathway and the influence of nitric oxide (NO) on it, **c** Wnt-Scl-LRP5/6 signalling pathway

The bone remodelling process was modelled following a previously published mathematical BCPM by Martin et al. (2019). This model considers the interactions between cells, the catabolic (RANK-RANKL-OPG) and anabolic (Wnt-Scl- LRP5/6) signalling pathways, together with the action of parathyroid hormone (PTH), nitric oxide (NO), transforming growth factor beta ($TGF-\upbeta$), and lastly considers the mechanobiological feedback on bone cells. The bone cell types (i.e. state variables) considered in the current model are: osteoblast precursor cells ($Ob_{\text{p}}$), active osteoblasts ($Ob_{\text{a}}$), osteoclast precursor cells ($Oc_{\text{p}}$), active osteoclasts ($Oc_{\text{a}}$) and osteocytes (Ot). The cell pools of uncommitted progenitor cells of both lineages ($Ob_{\text{u}}$, $Oc_{\text{u}}$) were assumed constant:

1 $${\frac{d \, {\text{Ob}_\text{p}}}{dt}} = D_{\text{Ob}_\text{u}} \cdot \text{Ob}_\text{u} \cdot \uppi_{\text{act,Ob}_\text{u}}^{\text{TGF-}\upbeta} - D_{\text{Ob}_\text{p}} \cdot \text{Ob}_\text{p} \cdot \uppi_{\text{rep,Ob}_\text{p}}^{\text{TGF-}\upbeta}  + {P_{\text{Ob}_\text{p}}} \cdot {\text{Ob}_\text{p}} \cdot {\uppi_{\text{act,Ob}_\text{p}}^{\text{Wnt}}} $$

2 $$\frac{d \,\text{Ob}_\text{a}}{dt} = D_{\text{Ob}_\text{p}} \cdot {\text{Ob}_\text{p}} \cdot \uppi_{\text{rep,Ob}_\text{p}}^{\text{TGF-}\upbeta} - \Delta_{\text{Ob}_\text{a}} \cdot {\text{Ob}_\text{a}}$$

3 $$\frac{d \, {\text{Oc}_\text{p}}}{dt} = D_{\text{Oc}_\text{u}} \cdot {\text{Oc}_\text{u}} \cdot \uppi_{\text{act,Oc}_\text{u}}^{\text{RANKL}} - D_{\text{Oc}_\text{p}} \cdot {\text{Oc}_\text{p}} \cdot \uppi_{\text{act,Oc}_\text{p}}^{\text{RANKL}}$$

4 $$\frac{d \, \text{Oc}_\text{a}}{dt} = D_{\text{Oc}_\text{p}} \cdot {\text{Oc}_\text{p}} \cdot \uppi_{\text{act,Oc}_\text{p}}^{\text{RANKL}} - A_{\text{Oc}_\text{a}} \cdot {\text{Oc}_\text{a}} \cdot \uppi _{\text{act, Oc}_\text{p}}^{\text{TGF-}\upbeta }$$

5 $$\frac{d\,\text{Ot}}{dt}=\eta \;\frac{d\,f_{bm}}{dt}$$

where $D_{\text{Ob}_{\text{u}}}$, $D_{\text{Ob}_{\text{p}}}$, $D_{\text{Oc}_{\text{u}}}$ and $D_{\text{Oc}_{\text{p}}}$ are the differentiation rates of $Ob_{\text{u}}$, $Ob_{\text{p}}$, $Oc_{\text{u}}$ and $Oc_{\text{p}}$, respectively; $A_{\text{Oc}_{\text{a}}}$ is the apoptosis rate of $Oc_{\text{a}}$ and ${\Delta_{\text{Ob}_\text{a}}}$ is the rate of clearance of active osteoblasts through apoptosis or differentiation into osteocytes. The variables ${\uppi_{\text{act,Ob}_{\text{u}}}^{\text{TGF-}\upbeta}}$, ${\uppi_{\text{rep,Ob}_\text{p}}^{\text{TGF-}\upbeta}}$ and ${\uppi_{\text{act, Oc}_\text{p}}^{\text{TGF-}\upbeta}}$ represent activator and repressor functions related to the binding of $TGF-\upbeta$ to its receptor. Similarly, ${\uppi_{\text{act,Oc}_\text{u}}^{\text{RANKL}}}$ and ${\uppi_{\text{act,Oc}_\text{p}}^{\text{RANKL}}}$ are the activator functions related to the RANK-RANKL binding. Finally, $P_{\text{Ob}_\text{p}}$ is the proliferation rate of $Ob_{\text{p}}$, a process which is mediated by the Wnt signalling pathway through the activator function $\pi_{\text{act,Ob}_{\text{p}}}^{\text{Wnt}}$ and is described in detail in Supplementary Material along with other features of the model. Model parameters of the cell population model are given in Table 2 of Supplementary Material. A schematic representation of the bone cell population model is shown in Fig. 1.

Equation (5) establishes that the population of osteocytes varies proportional to the bone matrix fraction $f_{bm}$, given that the density of osteocytes is constant within the bone matrix, $\eta$, is assumed constant, as done in (Martin et al. 2019). Finally, the variation of bone matrix fraction is obtained through the balance between resorbed and formed bone tissue:

6 $$\frac{{df_{{bm}} }}{{dt}} = - k_{{{\text{res}}}} \cdot {\text{Oc}}_{{\text{a}}} + k_{{{\text{form}}}} \cdot {\text{Ob}}_{{\text{a}}}$$

where $k_\text{res}$ and $k_\text{form}$ are, respectively, the rates of bone resorption and osteoid formation (see Table 2 of Supplementary Material).

### Competitive RANK-RANKL-OPG binding

The RANK-RANKL-OPG pathway includes the competitive binding of OPG to RANKL and RANKL to RANK. Following (Martin et al. 2019), the concentrations of OPG, RANK and RANKL are given by the following equations:

7 $$\begin{aligned} \displaystyle [\text{OPG}] = \frac{P_{\text{OPG}}}{\tilde{D}_{\text{OPG}}+\frac{\tilde{D}_{\text {OPG-RANKL}} \, [\text{RANKL}]}{K_{\text {OPG-RANKL}}}} \end{aligned}$$

8 $$[{\text{RANK}}] = \frac{{N_{{{\text{OC}}_{{\text{p}}} }}^{{{\text{RANK}}}} \cdot {\text{OC}}_{{\text{p}}} }}{{1 + \frac{{[{\text{RANKL}}]}}{{K_{{{\text{RANK - RANKL}}}} }}}}$$

9 $$\begin{gathered} [{\text{RANKL}}] = P_{{{\text{RANKL}}}} \cdot \left[ {\tilde{D}_{{{\text{RANKL}}}} + \frac{{\tilde{D}_{{{\text{RANK - RANKL}}}} }}{{K_{{{\text{RANK - RANKL}}}} }} \cdot [{\text{RANK}}]} \right. \hfill \\ \quad \quad \quad \quad \left. { + \frac{{\tilde{D}_{{{\text{OPG - RANKL}}}} }}{{K_{{{\text{OPG - RANKL}}}} }} \cdot [OPG] + \frac{{\tilde{D}_{{{\text{RANKL - Dmab}}}} }}{{K_{{{\text{RANKL - Dmab}}}} }} \cdot [{\text{Dmab}}]_{{{\text{BC}}}} } \right]^{{ - 1}} \hfill \\ \end{gathered}$$

$\tilde{D}_{X}$ and $\tilde{D}_{X-Y}$ are the degradation rates of the factor X and the complex X-Y, respectively; $K_{X-Y}$ is the dissociation constant of the complex *X*-*Y* and $N_{{{\text{OC}}_{{\text{p}}} }}^{{{\text{RANK}}}}$ is the number of RANK receptors per osteoclast precursor.${\text{[Dmab]}}_{{{\text{BC}}}}$ is the concentration of denosumab in the bone compartment (See Supplementary Material to learn more about the pharmacokinetics of the drug) and it is set to zero when we analyse the effect of age and the oestrogen deficiency alone, but it is different to zero when we simulate the administration of denosumab treatment. $P_\text{OPG}$ is the production rate of OPG by active osteoblasts:

10 $$P_{{{\text{OPG}}}} = \beta _{{{\text{OPG,Ob}}_{{\text{a}}} }} \pi _{{{\text{rep,Ob}}_{{\text{a}}} }}^{{{\text{PTH}}}} {\text{Ob}}_{{\text{a}}} \left( {1 - \frac{{[{\text{OPG}}]}}{{[{\text{OPG}}_{{\max }} ]}}} \right)$$

where $\beta _{{{\text{OPG,Ob}}_{{\text{a}}} }}$ is the OPG production rate, $\pi _{{{\text{rep,Ob}}_{{\text{a}}} }}^{{{\text{PTH}}}} {\text{Ob}}_{{\text{a}}}$ is the repressor function that quantifies the effect of PTH on the production of OPG and [OPG_max_] is the saturation concentration of OPG above which no further production takes place. To evaluate $P_{{{\text{RANKL}}}}$, the RANKL production rate of Eq. (9), we have assumed that RANKL is expressed by osteocytes and osteoblast precursors following experimental evidence (Nakashima et al. 2011; Xiong et al. 2015) and then:

11 $$\begin{gathered} P_{{{\text{RANKL}}}} = \beta _{{{\text{RANKL}},{\text{OB}}_{{\text{p}}} }} \cdot \pi _{{{\text{act/rep,RANKL}}}}^{{{\text{PTH,NO}}}} \cdot \left( {1 - \frac{{[{\text{RANKL}}]_{{{\text{tot}}}} }}{{[{\text{RANKL}}]_{{{\text{max}}}} }}} \right) \cdot {\text{OB}}_{{\text{p}}} \hfill \\ \quad \quad \quad + \beta _{{{\text{RANKL,Ot}}}} \cdot \pi _{{{\text{act,RANKL}}}}^{{{\text{dam}}}} \cdot \left( {1 - \frac{{[{\text{RANKL}}]_{{{\text{tot}}}} }}{{[{\text{RANKL}}]_{{{\text{max}}}} }}} \right) \cdot Ot + P_{{{\text{RANKL}}}}^{{{\text{ED}}}} \hfill \\ \end{gathered}$$

where $\beta _{{{\text{RANKL,OB}}_{{\text{p}}} }}$ and $\beta _{{{\text{RANKL,Ot}}}}$ are the RANKL production rate of osteocytes and osteoblast precursors, respectively; $\pi _{{{\text{act/rep,RANKL}}}}^{{{\text{PTH,NO}}}}$ is a co-regulatory function that takes into account the up-regulation of RANKL transcription by the parathyroid hormone (PTH) and its inhibition by nitric oxide (NO) (Martin et al. 2019) and $\pi _{{{\text{act,RANKL}}}}^{{{\text{dam}}}}$ is an activator function accounting for the upregulation of RANKL expression by osteocytes due to microstructural damage ( Martínez-Reina et al. (2021b)) (see the details of both regulatory functions in Supplementary Material). $P_{\text{RANKL}}^{\text{ED}}$ is the RANKL production due to oestrogen deficiency. Finally, [RANKL]_max_ is the saturation concentration of RANKL above which no further expression takes place and [RANKL]_tot_ is the total concentration of RANKL (bound and free) and is defined as follows:

12 $$[\text{RANKL}]_{\text{tot}}=[\text{RANKL}] \cdot \left( {1 + \frac{{[{\text{RANK}}]}}{{K_{{{\text{RANK - RANKL}}}} }} + \frac{{[{\text{OPG}}]}}{{K_{{{\text{OPG - RANKL}}}} }} + \frac{{[{\text{Dmab}}]_{{{\text{BC}}}} }}{{K_{{{\text{RANKL - Dmab}}}} }}} \right)$$

The activation functions shown in Eq. (3) and in Eq. (4) have the following structure:

13 $$\begin{aligned} \displaystyle  {\uppi _{\text{act,X}}^{\text{RANKL}}} = \frac{\text {[RANKL]}}{K_{\text{act,X}^{\text{RANKL}}+[\text{RANKL}]}} \qquad \text {with X =} \,  {\text{Oc}_\text{u},\, \text{Oc}_\text{p}} \end{aligned}$$

Through $\pi _{{{\text{act,Oc}}_{{\text{u}}} }}^{{{\text{RANKL}}}}$ and $\pi _{{{\text{act,Oc}}_{{\text{p}}} }}^{{{\text{RANKL}}}}$, the RANK-RANKL-OPG pathway controls the differentiation of uncommitted osteoclast progenitors and osteoclasts precursors, respectively. In this way, if there is an imbalance in that pathway, it will develop in osteoporosis or in osteopenia depending on how severe the oestrogen deficiency is.

### Competitive Wnt-sclerostin-LRP5/6 binding

An additional effect of age has been simulated by an increase in serum sclerostin. This has been modelled by including a sclerostin external production term, and for this reason, the main equations that correspond to the sclerostin production are provided below. Consulting Supplementary Material is advised for a better understanding of this and other equations referring to the Wnt-Scl-LRP5/6 competitive binding. The production of sclerostin is given by the next equation:

14 $$\begin{aligned} \displaystyle P_\text{Scl,b} + P_\text{Scl,d} = \tilde{D}_{\text{Scl}} \, \text {[Scl]} + \tilde{D}_{\text {Scl-LRP5/6}} \, \text {[Scl-LRP5/6]} \end{aligned}$$

where $\tilde{D}_{\text{Scl}}$ and $\tilde{D}_{\text{Scl-LRP5/6}}$ are the degradation rates of sclerostin and the sclerostin-LRP5/6 complex, respectively. The endogenous production of sclerostin by osteocytes is:

15 $$\begin{aligned} \displaystyle P_{\text{Scl,b}} = \beta_{Scl,Ot} \, \uppi_{rep,Scl}^{\left| \varepsilon \right|_{max}} \, Ot \, \left(1-\frac{[Scl]}{[Scl]_{max}}\right)\end{aligned}$$

where $\beta _\text{Scl,Ot}$ and ${[\text{Scl}]_{\text{max}}}$ are, respectively, the sclerostin production rate and its maximum concentration. The production of sclerostin by osteocytes is downregulated by the mechanical stimulus through the repressor function $\uppi_{\text{rep,Scl}}^{\left| \varepsilon\right|_{max}}$ (see Supplementary Material).

The external dosage of sclerostin was modelled by using a bilinear function, and it is shown in Eq. 20 later on.

### Regulatory role of $TGF-\beta$

$TGF-\upbeta$ is stored in the bone matrix and released during resorption by osteoclasts. Its concentration is calculated following (Pivonka et al. 2008):

16 $$\begin{aligned} \displaystyle [\text{TGF}-\upbeta ] = \frac{\alpha _{\text{TGF}-\upbeta } \; k_{res} \; Oc_a }{\tilde{D}_{\text{TGF}-\upbeta}} \end{aligned}$$

where $\alpha _{\text{TGF}-\upbeta }$ is the concentration of $TGF-\upbeta$ in bone matrix and $\tilde{D}_{\text{TGF}-\upbeta }$ is the $TGF-\upbeta$ degradation rate. The concentration of $TGF-\upbeta$ is used to define the activator/repressor functions that appear in Eqs. (1), (2) and (4):

17 $$\begin{aligned}  {\uppi _{\text{act,Ob}_\text{u}}^{\text{TGF}-\upbeta }}&=  {\uppi _{\text{act, Oc}_\text{p}}^{\text{TGF}-\upbeta }} = \frac{[\text{TGF}-\upbeta ]}{K_{{\text{act}}^{\text{TGF}-\upbeta }} + [\text{TGF}-\upbeta ]} \end{aligned}$$

18 $$\begin{aligned} {\uppi_{\text{rep,Ob}_\text{p}}^{\text{TGF}-\upbeta}}&= \frac{K_{\text{rep}}^{TGF-\upbeta}}{K_{\text{rep}}^{\text{TGF}-\upbeta}+[\text{TGF}-\upbeta]}\end{aligned}$$

These functions control the upregulation of the differentiation of ${\text{Ob}_{\text{u}}}$ into ${\text{Ob}_{\text{p}}}$, the upregulation of osteoclast apoptosis and the downregulation of the differentiation of ${\text{Ob}_{\text{p}}}$ into ${\text{Ob}_{\text{a}}}$. Despite the latter, $TGF-\upbeta$ increases the pool of ${\text{Ob}_{\text{p}}}$, leading to a delayed increase of ${\text{Ob}_{\text{a}}}$ concentration. This and the upregulation of osteoclast apoptosis give $TGF-\upbeta$ a global anabolic effect.

### Modelling the effect of ageing and menopause

We tested two ways of modelling the effect of ageing on bone turnover: (1) a decrease in the concentration of $TGF-\upbeta$ and (2) an increase in the production of sclerostin. Similarly, we tested three ways to take into account the effect of menopause by considering the pathways through which oestrogen affects bone remodelling: (1) an increased production of RANKL, (2) an increased responsiveness of osteoclast to RANKL and (3) a decreased production of OPG. The details of each are outlined below.

#### Decreased release of $TGF-\upbeta$ from bone matrix through resorption

The effect of ageing is modelled based on the experimental findings reported in Nicolas et al. (1994) and the approach suggested by Lemaire et al. (2004), i.e. by reducing the $TGF-\upbeta$ content in bone matrix, $\alpha _{\text{TGF}-\upbeta }$. In contrast to Lemaire et al. who modelled an abrupt decrease of $TGF-\upbeta$ concentration, in our model $TGF-\upbeta$ reduces continuously over time. Given that Riggs et al. (2004) reported a decrease in bone mass of vertebral trabecular bone starting at age $t_{\text{mat}}=20$ years, we have assumed that the effects begin at that age.

Nicolas et al. (1994) concluded that there is a decrease of $TGF-\upbeta$ over time; however, when they compared two groups of age (20–29 years and 50–59 years), they could not find significant differences. Other authors have recently found more conclusive results that would confirm the negative correlation between age and TGF-$\upbeta$ content in both serum (Okamoto et al. 2005) and bone (Pfeilschifter et al. 1998). In the latter case, there is a clear reduction of $TGF-\upbeta$ content within the interval between 30 and 50 years and there is no difference after 50 years of age. Based on these findings, we have used a bilinear function to simulate the decrease of $TGF-\upbeta$:

19 $$\begin{aligned} \alpha _{\text{TGF}-\upbeta } (t) = \left\{ \begin{array}{ll} \displaystyle \alpha _{\text{TGF}-\upbeta ,0} &{} \quad \text {for} \;\; \text{TSSM}<0 \\ \alpha _{\text{TGF}-\upbeta ,0} - k_1 \; \text{TSSM} &{} \quad \text {for} \;\; 0 \le \text{TSSM} < T_1 \\ \alpha _{\text{TGF}-\upbeta ,0} - k_1 \; T_1 - k_2 \, (\text{TSSM}-T_1) &{} \quad \text {for} \;\; T_1 \le \text{TSSM} \end{array} \right. \end{aligned}$$

where $\text{TSSM} = t - t_{\text{mat}}$ is expressed in days and $t_{\text{mat}}$ is the age at which bone mass begins to decline. Riggs et al. (2004) measured the descent from 20 years onwards, the age of skeletal maturity. Thus, for the sake of brevity, that difference has been named TSSM, after Time Since Skeletal Maturity, though no direct relation has been established between skeletal maturity and the beginning of bone mass loss. The slopes k_1_ and k_2_ measure the rate of decay of the concentration of $TGF-\upbeta$ in the bone matrix over time. We have assumed that this rate of decay can vary after T_1_ days elapsed since $t_{\text{mat}}$. T_1_ will be adjusted along with k_1_ and k_2_ to fit the observed decrease in bone mass with age. It must be noted that we imposed a constraint in the constants k_1_, k_2_ and T_1_ so that $\alpha _{\text{TGF}-\upbeta } (95 \text{yr}) \ge 0.9$. This was done so to avoid the total loss of bone that lower values of $\alpha _{\text{TGF}-\upbeta }$ systematically produced in ARO+PMO simulations. This constraint is also justified by a recent work (Calvo-Gallego et al. 2023) in which we have shown that $TGF-\upbeta$ plays a fundamental role in the coordination of the sequence resorption-reversion-formation of the BMU and therefore its value cannot be very small.

#### Increase of the production of sclerostin

It was assumed in Martin et al. (2019) that the increased serum concentration of sclerostin was an effect of menopause. However, several studies (Ardawi et al. 2011; Mödder et al. 2011) suggested that this must be an effect of age since the increasing sclerostin levels are already observed in premenopausal women (Ardawi et al. 2011) and also in men (Mödder et al. 2011). Based on these studies, we have considered an increase in the external production of sclerostin, $P_{\text{Scl,d}}$. Roforth et al. (2014) suggested that ageing could be associated with a reduced clearance of sclerostin from the circulation, but also with increased sclerostin production by non-skeletal sources. Indeed, circulating sclerostin may be derived not only from osteocytes (Ke et al. 2012), but also from several other sources, including myeloid cells (Kusu et al. 2003; Pederson et al. 2009; Ota et al. 2013), vascular smooth muscle cells (Zhu et al. 2011), and the kidney (Ke et al. 2012). We have opted for an external production of sclerostin and not considered a reduction in the clearance through $\tilde{D}_{\text{Scl}}$, but both effects would lead to the same sclerostin serum concentration.

20 $$\begin{aligned} P_{\text{Scl,d}} (t) = \left\{ \begin{array}{ll} \displaystyle 0 &{} \quad \text {for} \;\; \text{TSSM}<0 \\ k_3 \; \text{TSSM} &{} \quad \text {for} \;\; 0 \le \text{TSSM} < T_2 \\ k_3 \; T_2 + k_4 \, (\text{TSSM}-T_2) &{} \quad \text {for} \;\; T_2 \le \text{TSSM} \end{array} \right. \end{aligned}$$

where the slopes $k_3$ and $k_4$ measure the rate of increase in the external production of sclerostin. We have assumed that the rate of external sclerostin production can vary after $T_2$ days elapsed since $t_{\text{mat}}$.

Sclerostin inhibits the proliferation of $Ob_p$ and eventually leads to a lower population of $Ob_a$, so decreasing the rate of formation of new tissue in remodelling events and consequently reducing the BMD if its levels are increased.

#### Increased production of RANKL

We will consider an external RANKL production due to oestrogen deficiency, $P_{\text{RANKL}}^{\text{ED}}$, as done in previous studies (Scheiner et al. 2014; Martínez-Reina et al. 2021b) and we will assume here that $P_{\text{RANKL}}^{\text{ED}}$ increases over time after menopause following a bilinear function defined through the parameters $k_5$, $k_6$ and $T_3$ and adjusted later on:

21 $$\begin{aligned} P_{\text{RANKL}}^{\text{ED}}(t) = \left\{ \begin{array}{ll} 0 &{} \quad \text {for} \;\; \text{TSM}< 0 \\ k_5 \; TSM &{} \quad \text {for} \;\; 0 \le \text{TSM} < T_3 \\ k_5 \; T_3 + k_6 \, (\text{TSM} - T_3) &{} \quad \text {for} \;\; T_3 \le \text{TSM} \end{array} \right. \end{aligned}$$

where $\text{TSM} = t - t_{\text{meno}}$ is the time elapsed since menopause and expressed in days. Following (Nordin et al. 1990), we have assumed here $t_{\text{meno}}=49$ years. This external production of RANKL would be in accordance with the conclusions of Eghbali-Fatourechi et al. (2003) about the increased expression of RANKL by stromal cells and lymphocytes with a decreased oestrogen production. This production would increase the RANKL levels and promote the differentiation of $Oc_a$, imbalancing bone remodelling events in favour of resorption and thus leading to bone loss.

#### Increased responsiveness of osteoclasts to RANKL

Shevde et al. (2000) specifically stated that the increased responsiveness of cells of the osteoclastic lineage to RANKL following oestrogen decay is exclusive to the differentiation of osteoclast precursors into active osteoclasts. Thus, in the ${\uppi }$ functions related to RANKL (see Eq. 13) we will only decrease $K_{\text{act,Oc}_\text{p}}^{\text{RANKL}}$ with a bilinear function of time and we will keep $K_{\text{act,Oc}_\text{u}}^{\text{RANKL}}$ constant:

22 $$\begin{aligned} K_{\text{act,Oc}_\text{p}}^{\text{RANKL}} (t) = \left\{ \begin{array}{ll} K_{\text{act,Oc}_\text{p},0}^{\text{RANKL}} &{} \quad \text{for} \;\; TSM< 0 \\ K_{\text{act,Oc}_\text{p},0}^{\text{RANKL}}- k_7 \; \text{TSM} &{} \quad \text {for} \;\; 0 \le \text{TSM} < T_4 \\ K _{\text{act,Oc}_\text{p},0}^{\text{RANKL}} - k_7 \; T_4 - k_8 \, (\text{TSM}-T_4) &{} \quad \text {for} \;\; T_4 \le \text{TSM} \end{array} \right. \end{aligned}$$

where the parameters k_7_, k_8_, T_4_and will be adjusted to obtain the evolution of bone mass loss after menopause.

Both an increased production of RANKL, such as the one considered in Sect. 2.5.3, and an increased responsiveness to RANKL, through the reduction of $K_{\text{act,Oc}_\text{p}}^{\text{RANKL}}$, give rise to an increasing value of the function ${\uppi _{\text{act,X}}^{\text{RANKL}}}$ in Eq. (13) and consequently increase the population of resorbing osteoclasts.

#### Reduced production of OPG by osteoblasts

According to Hofbauer et al. (1999); Viereck et al. (2003) we will decrease the production of OPG by osteoblasts through the factor $\beta _{\text{OPG,Ob}_\text{a}}$ in Eq. (10) as follows:

23 $$\begin{aligned} \beta _{\text {OPG,Ob}_\text{a}} (t) = \left\{ \begin{array}{ll} \beta _{\text {OPG,Ob}_\text{a,0}} &{} \quad \text {for} \;\; \text{TSM}< 0 \\ \beta _{\text {OPG,Ob}_{\text{a},0}} - k_9 \; \text{TSM} &{} \quad \text {for} \;\; 0 \le \text{TSM} < T_5 \\ \beta _{\text {OPG,Ob}_{\text{a},0}} - k_9 \; T_5 - k_{10} \; (TSM - T_5) &{} \quad \text {for} \;\; T_5 \le \text{TSM} \end{array} \right. \end{aligned}$$

where, again, the parameters $k_9$, $k_{10}$, and $T_5$ will be adjusted to fit the bone mass loss after menopause. Decreasing $\beta _{\text{OPG,Ob}_\text{a}}$ results in dropping levels of OPG and a lower possibility of RANKL-OPG binding. Therefore, more RANKL is free to bind to RANK, thus promoting $Oc_a$ differentiation and enhancing bone resorption.

### Adjustment of parameters

The nominal value $\alpha_{\text{TGF}-\upbeta ,0}$ was taken from a previous version of the model (Martin et al. 2019), whereas $K_{\text{act,Oc}_{\text{p},0}}^{\text{RANKL}}$ and $\beta_{\text{OPG,Ob}_{\text{a,}0}}$ were readjusted in this work based on the values reported in the literature (see Supplementary Material).

Next, we adjusted the slopes k_i_ with i=1,2,3,4 and the times T_j_ with j=1,2 to model the effect of ageing on bone loss, so to reproduce the results obtained by Riggs et al. (2004) for men. Once the effect of ageing was adjusted, we added the effect of oestrogen deficiency to reproduce the results obtained for women and adjusted the slopes k_i_ with $i=5,\ldots ,10$ and the times T_j_ with j=3,4,5. A summary of the simulations performed to adjust the parameters is given in Table 1.

**Table 1:** Summary of the simulations performed to adjust the effect of age and oestrogen deficiency

Simulation #	Disease state	Effects considered	Variables assumed constant	Variables to adjust	Validation
S1	ARO	Decrease in TGF$-\upbeta$	$\alpha _{\text{TGF}-\upbeta ,0}$	$k_1$,$k_2$,$T_1$	Men in Riggs et al. (2004)
S2	ARO	Increase in Scl production	–	$k_3$,$k_4$,$T_2$	Men in Riggs et al. (2004)
S3	ARO	Decr. in TGF$-\upbeta$ and incr. in Scl	$\alpha _{\text{TGF}-\upbeta ,0}$	$k_1$,$k_2$,$T_1$	Men in Riggs et al. (2004)
				$k_3$,$k_4$,$T_2$	
S4	ARO+PMO	Age + RANKL production	$k_1$,$k_2$,$T_1$	$k_5$,$k_6$,$T_3$	Women in Riggs et al. (2004)
			$k_3$,$k_4$,$T_2$		
S5	ARO+PMO	Age + Increased responsiveness to RANK	$k_1$,$k_2$,$T_1$	$k_7$,$k_8$,$T_4$	Women in Riggs et al. (2004)
			$k_3$,$k_4$,$T_2$		
			$$K_{\text{act,Oc}_{\text{p}},0}^{\text{RANKL}}$$		
S6	ARO+PMO	Age + Decrease in OPG production	$k_1$,$k_2$,$T_1$	$k_9$,$k_{10}$,$T_5$	Women in Riggs et al. (2004)
			$k_3$,$k_4$,$T_2$		
			$$\beta _{\text{OPG,Ob}_{\text{a},0}}$$		
S7	ARO+PMO	Age + All effects of oestrogen deficiency combined	$k_1$,$k_2$,$T_1$	$k_5$,$k_6$,$T_3$	Women in Riggs et al. (2004)
			$k_3$,$k_4$,$T_2$	$k_7$,$k_8$,$T_4$	
			$\beta_{\text{OPG,Ob}_{\text{a}},0}$ $K_{\text{act,Oc}_{\text{p}},0}^{\text{RANKL}}$	$k_9$,$k_{10}$,$T_5$	

**Fig. 2:**
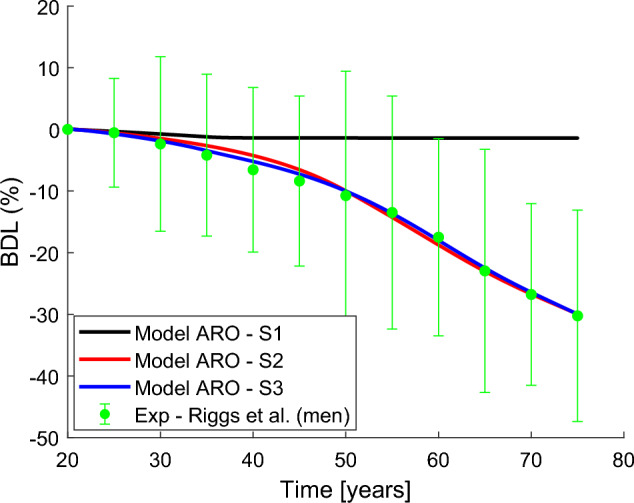
Osteoporosis disease system modelling in men: bone density loss (%) in trabecular bone of vertebral body versus time adjusted for age (years). (1) Only including the decrease in TGF-$\beta$ concentration of bone matrix (black), (2) only including the increasing production of sclerostin (red) and (3) including both effects (blue). The curves are compared with the experimental results from Riggs et al. (2004) (green dots and error bars) showing age-related bone loss in men. (Color figure online)

**Fig. 3:**
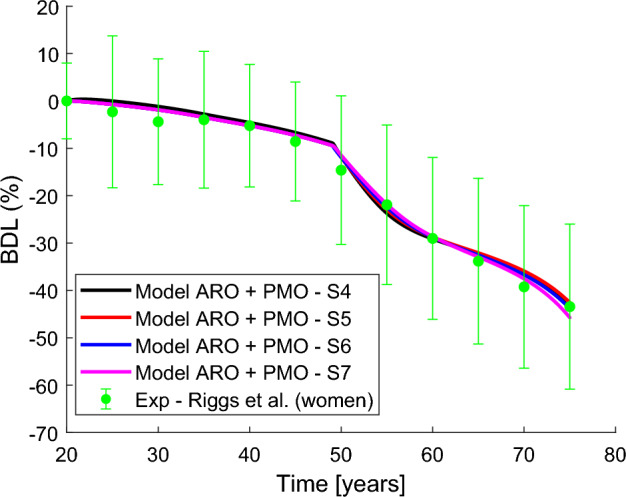
Osteoporosis disease system modelling in women: bone density loss (%) in trabecular bone of vertebral body versus time adjusted for age (years), when the effect of ageing is considered as in S3 and the effect of oestrogen deficiency is considered as follows. (1) Only including the increase of RANKL production (black), (2) only including the increasing responsiveness to RANK (red), (3) only including the decrease in OPG production (blue) and (4) including the three effects (green). The curves are compared with the experimental results from Riggs et al. (2004) (green dots and error bars) showing age-related bone loss in women. (Color figure online)

In their cross-sectional study (Riggs et al. 2004) used QCT to measure vBMD at different bone sites, in a large population of individuals of both sexes (373 women and 323 men) and over a wide age range (20–97 years), which makes this study very appropriate to validate our results. We have selected their data corresponding to the lumbar spine and used the density vs. age regression curves that they provided for each sex. We have located on these curves a series of equispaced points every 5 years (green points in Figs. 2 and 3). For clarity, we will show in those figures the error bars corresponding to ±1 standard deviation instead of the point cloud provided by Riggs et al. (2004). Taking as a reference the average density at 20 years of age, we have calculated the percentage bone density loss with respect to that age, using:

24 $$\begin{aligned} \displaystyle \text{BDL}_i\;(\%) = \frac{\rho (t_i)-\rho (20)}{\rho (20)} \, \cdot 100 \end{aligned}$$

where $\rho (t_i)$ and $\rho (20)$ represent the density at age $t_i$ and 20 years, respectively. Also, taking the densities from the curves given by Riggs et al. (2004) or from our model, we can obtain, respectively, $\text{BDL}_i^R$ or $\text{BDL}_i^M$. Note that $\rho$ in BCPM model is equivalent to vBMD measured in experiments.

We adjusted the corresponding constants $k_i$ and $T_j$ of our model through the least squares method, by minimising the root mean square error (RMSE) between the points of Riggs et al. and those estimated with our model:

25 $$\begin{aligned} \text{RMSE} = \sqrt{\frac{\sum _{i=1}^n \big ( \text{BDL}_i^R - \text{BDL}_i^M \big )^2}{n}} \end{aligned}$$

where *n* is the number of time points we have selected on the regression curves of Riggs et al. Let us recall that a constraint was imposed on constants $k_1$, $k_2$ and $T_1$ on the simulations of ARO (S1 and S2), so that $\alpha _{\text{TGF}-\upbeta } (95 \text{yr}) \ge 0.9$

## Results

The results of the adjustments are summarised in Table 2 with the RMSE of the best adjustment. S1, S2 and S3 correspond to the adjustment of ARO and the remaining parameters to ARO+PMO.

**Table 2:** Summary of the simulations performed to adjust the effect of age and menopause

Simulation #	Adjusted parameters			RMSE
S1	$k_1=1.786 \cdot 10^{-5}$	$k_2=1.017\cdot 10^{-7}$	$T_1=5504$	14.54%
S2	$k_3=1.450\cdot 10^{-2}$	$k_4=7.5\cdot 10^{-3}$	$T_2=12780$	1.34%
S3	$k_1=1.797\cdot 10^{-5}$	$k_2=1.000\cdot 10^{-7}$	$T_1=5475$	0.67%
	$k_3=1.100\cdot 10^{-2}$	$k_4=5.004\cdot 10^{-4}$	$T_2=20072$	
S4	$k_5=1.600\cdot 10^{-2}$	$k_6=1.000\cdot 10^{-5}$	$T_3=100.2$	1.57%
S5	$k_7=1.000\cdot 10^{-1}$	$k_8=5.005\cdot 10^{-4}$	$T_4=185$	1.75%
S6	$k_9=5.000\cdot 10^3$	$k_{10}=4.957$	$T_5=254.9$	1.55%
S7	$k_5=3.446\cdot 10^{-4}$	$k_6=1.009\cdot 10^{-6}$	$T_3=154.4$	1.44%
	$k_7=3.875\cdot 10^{-4}$	$k_8=1.008\cdot 10^{-6}$	$T_4=137.8$	
	$k_9=7.979\cdot 10^2$	$k_{10}=3.243$	$T_5=143.3$	

The slopes $k_1$, $k_2$, $k_7$ and $k_8$ are given in pM/days, $k_3$, $k_4$, $k_5$ and $k_6$ are given in pM/days^2^, $k_9$, $k_{10}$ are given in pM OPG/pM Cell/days^2^ and the times $T_j$ in days. RMSE calculated against data given by Riggs et al. (2004)

Figure 2 compares the best adjustments obtained for each simulation of ARO with the clinical results obtained by Riggs et al. (2004) for men. If only a decrease in the content of $TGF-\upbeta$ in bone matrix is considered (S1), little bone density loss is observed (black), resulting in a large mean error (see Table 2). There is a slight initial fall of bone density, but it soon stabilises around age 40.

If only sclerostin production by non-skeletal sources is considered (S2), there is a marked and sustained drop in bone density (red), which closely resembles the clinical results (mean error 1.34%). This error was reduced, though only slightly, when both effects (sclerostin and $TGF-\upbeta$, S3) were considered (blue line) and the evolution of density adjusts better the clinical results, especially in middle ages (35 to 50). In the following, the constants adjusted in S3 will be adopted to simulate the effect of age in the combined ARO+PMO simulations.

Figure 3 compares the best adjustments obtained for the simulations of ARO+PMO with the clinical results obtained by Riggs et al. (2004) for women. It can be seen that all the cases lead to similar curves and produce similar errors (see Table 2). For simplicity and due to the similarity of the simulations of ARO+PMO, we will focus on the results of S4 from now on.

**Fig. 4:**
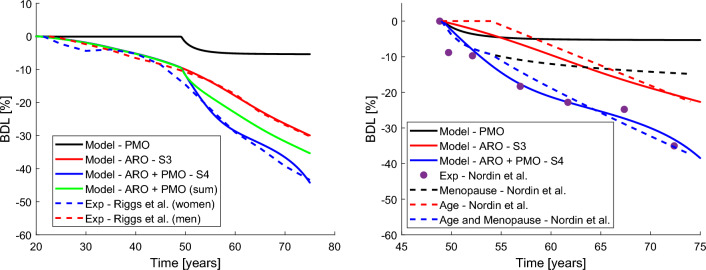
Comparison of the relative effects of age and menopause. BDL is measured from the beginning of the simulation (20 years of age) in the left plot, where the results are compared with the regression curves obtained by Riggs et al. (2004). BDL is measured from menopause (49 years of age) in the right plot, as done by Nordin et al. (1990) with whose results are compared. (1) Only the effect of oestrogen deficiency is considered (black). (2) Only the effect of age is considered (red). (3) The effects of age and oestrogen deficiency are considered jointly in the model (blue). (4) BDL curves black and red are summed in the green curve at the left. (Color figure online)

Figure 4 compares the separate effect of age and oestrogen deficiency and the combination of both. In the left plot, BDL is measured from the beginning of the simulation (20 years of age) and the results are compared with the regressions obtained by Riggs et al. (2004) (dashed lines). In the right plot, BDL is measured from menopause (assumed to be at 49 years of age) as done by Nordin et al. (1990) with whose results are compared (dashed lines for the regression and dots for the average values at certain ages).

Let us analyse first the left plot. If only the effect of oestrogen deficiency is considered in the simulation ($k_5$, $k_6$ and $T_3$ of S4 in Table 2 and no effect of age), no BDL is seen until menopause (black line). Then, an abrupt bone loss is seen during the first 5 years, followed by a stabilisation of that bone loss. If only the effect of age is considered ($k_1$ to $k_4$ plus $T_1$ and $T_2$ of S3 in Table 2), a continuous descent of bone density is obtained (red line), fitting very accurately the results of Riggs et al. (2004) for men. As shown before, the simulation of the combined effect of ageing and oestrogen deficiency (case S4) led to a very marked drop of bone mass right after menopause (blue line), which is much stronger than in the case where only oestrogen deficiency was considered and does not stabilise. Furthermore, the effects of ageing and menopause are coupled in the model to some extent, since the superposition of the BDL obtained with each separate effect (green line, which is the sum of the contributions of the red and black lines) underestimates the BDL when both effects are modelled jointly (blue line). In other words, the coupled effects of ageing and oestrogen deficiency manifest in a more pronounced disease state.

In their cross-sectional study Nordin et al. measured the evolution of forearm mineral density (FMD) with age in a cohort of 485 postmenopausal women of mean age 59± 0.3 yr (range 33–75) and for whom the age at menopause could be established (Nordin et al. 1990) (mean value 49.2±0.21 yr). These authors adjusted FMD decay using a linear term to account for age and a logarithmic term to account for menopause through YSM (black dashed line). They assumed menopausal age at 49 years and that no bone loss is due exclusively to age until 54 years. Therefore, they proposed the following equations for ages over 49:

26 $$\begin{array}{*{20}l} {{\text{FMD}}\left( {\frac{{{\text{mg}}}}{{{\text{cm}}^{{\text{3}}} }}} \right) = 451 - 16 \cdot {\text{ln}}({\text{YSM}})} \hfill & {{\text{if age}} \le {\text{54 years}}} \hfill \\ {{\text{FMD}}\left( {\frac{{{\text{mg}}}}{{{\text{cm}}^{{\text{3}}} }}} \right) = 451 - 5.3 \cdot ({\text{age}} - 54) - 16 \cdot {\text{ln}}({\text{YSM}})} \hfill & {{\text{if age}}} > {{\text{54 years}}} \hfill \\ \end{array}$$

BDL can be calculated from 49 years onwards using this formula, as well as the BDL corresponding to the age and menopause terms. The three curves of BDL so calculated are plotted in Fig. 4 (right) in dashed lines, in black (YSM), red (age) and blue (total), respectively. The BDL obtained with our simulations is also shown in solid lines, along with the points obtained from the mean FDM at given ages.

**Fig. 5:**
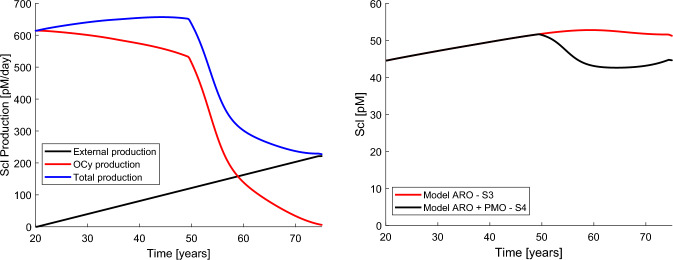
Left: different sources of sclerostin production. (1) Sclerostin produced by osteocytes (red) as obtained in the simulation S4. (2) External supply via serum, e.g. non-osseous cells (black). This law was adjusted in the simulation S3 and assumed fixed for the rest of simulations. (3) Total production of sclerostin (blue), sum of (1) and (2). Right: evolution of the concentration of sclerostin in bone: (1) in ARO (red, simulation S3) and (2) in ARO+PMO (black, simulation S4). (Color figure online)

The effect of sclerostin has been shown here to be key in predicting bone loss, due to ageing and to the variations induced by oestrogen deficiency. For this reason, we show the supply of sclerostin to bone in Fig. 5 (left). The external supply via non-osseous cells was adjusted in the simulation S3 to fit the effect of ageing in bone loss and this law was subsequently imposed in the simulations of ARO+PMO. The production of sclerostin by osteocytes depends on that external supply and the evolution corresponding to simulation S4 is plotted in red, along with the total production in blue, which is the sum of both contributions. Figure 5 (right) shows the temporal evolution of the concentration of sclerostin in bone, which is slowly increasing with age until menopause when it falls during roughly 10 year to stabilise afterwards.

**Fig. 6:**
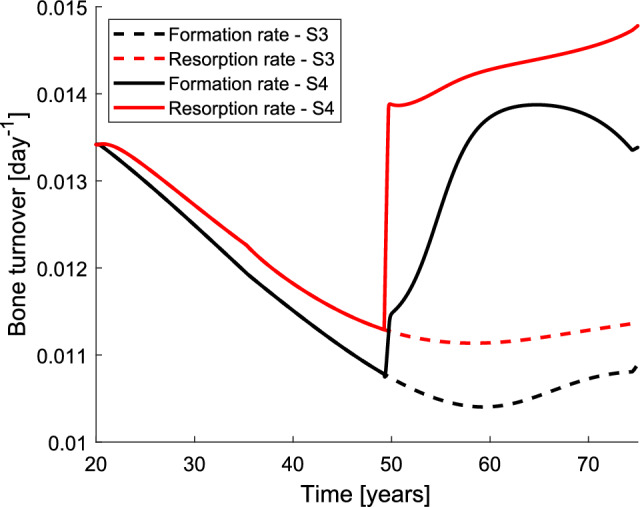
Evolution of the predicted bone resorption rate (red) and bone formation rate (black): in men (dashed lines), where only the effect of age is considered, and in women (solid lines), where both age and oestrogen deficiency are considered. Note that before menopause, when only ageing has an effect, the solid and dashed lines coincide. (Color figure online)

Figure 6 shows the evolution of bone resorption and bone formation rates over time, distinguishing the simulations that only consider the effect of age (dashed lines) and would correspond to men from those corresponding to women in which the effect of oestrogen deficiency is also considered (solid lines).

**Fig. 7:**
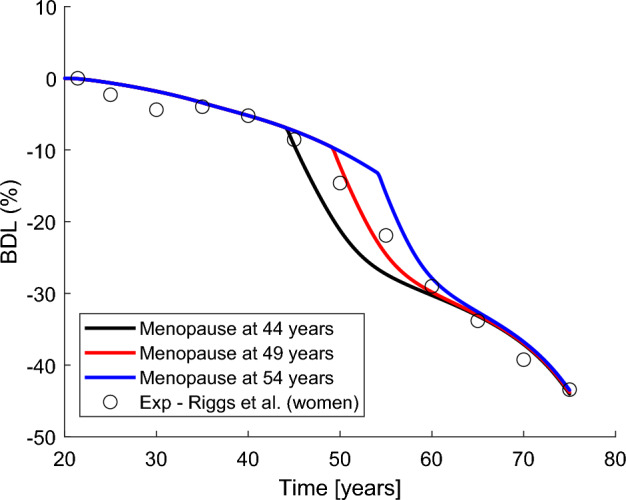
Comparison of ARO+PMO simulations for different ages at menopause: 44 (black), 49 (red) and 54 (blue) years. (Color figure online)

Figure 7 analyses the influence of the age at menopause in the evolution of BDL, by comparing the nominal case analysed before (49 years) with the cases of early and late menopause (44 and 54 years, respectively). Our simulations predict a shift from the ageing curve at the onset of menopause and a convergence of the curves in the long-term.

**Fig. 8:**
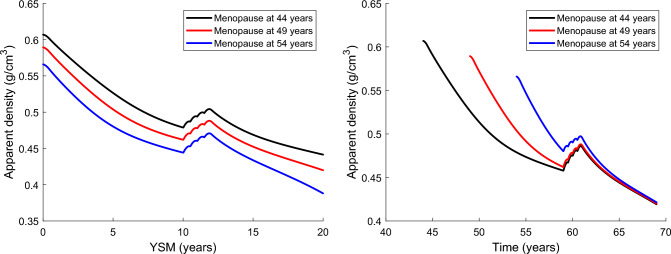
Predicted evolution of the apparent density of trabecular bone for women who went through menopause at different ages and were treated with denosumab (60Q6) for two years. Left, the treatment is started 10 years after menopause. Right, the treatment is started at the age of 59

In Fig. 8, we compare the effectiveness of the WHO-approved Dmab treatment (60 mg of Dmab injected every 6 months, 60Q6) in patients with different menopause ages. Doing so, we highlight the importance of distinguishing both timelines, age and YSM, in the analysis of the treatment efficacy. This distinction contrasts with previous studies which did not take into account age and consequently could only analyse the effect of YSM (Martínez-Reina and Pivonka 2019; Martínez-Reina et al. 2021a, b; Calvo-Gallego et al. 2022). In Fig. 8 left the treatment commences 10 years after menopause, i.e. at different ages and we note that the bone density gain is similar for the three cases in absolute terms (around 0.025 g/cm^3^ in two years). In the right figure, the treatment commences at the age of 59 regardless of the age at menopause and the bone density gain is similar for normal (49 years) or early menopause (44 years), respectively, 5.7% and 6.2% of bone density gain since the beginning of the treatment. However, the drug is not as effective for women with late menopause (54 years), for whom the treatment has been started only 5 years after menopause, probably too soon, and bone density gain is only 3.6%.

## Discussion

In this paper we have presented a model of bone remodelling that is able to discern the effect on bone loss of ageing, during the whole life, and of oestrogen deficiency, occurring around menopause. It is important to consider both effects separately for an accurate in silico simulation of both, OP and treatment. Even in the design of the treatment, both effects should also be considered separately, as some of the treatments focus on counteracting the effect of menopause, such as the increase of RANKL in the case of Dmab, but are not able to counteract the effect of ageing and more importantly, the possible coupling between both effects.

The effects of both factors contribute to bone loss in women. In men, the drop in androgen levels has also been reported to diminish bone formation (Kasperk et al. 1990, 1997) and increase osteoclastogenesis (Bellido et al. 1995; Pilbeam and Raisz 1990) and the overall effect may be similar to that of menopause. However, this androgen decline is less pronounced but more sustained over time (Vermeulen 2000), just the opposite of the oestrogen decline around menopause. Moreover, it is not easy to identify it with an age range, as in menopause. Thus, the data from Riggs et al. (2004) for men do not show the sharp decline in bone mass that is seen in women around the age of 50.

The effect of ageing has been taken into account in previous works (Lemaire et al. 2004) by means of a decrease in the concentration of $TGF-\upbeta$ accumulated in bone matrix and following some clinical evidence (Nicolas et al. 1994; Okamoto et al. 2005; Pfeilschifter et al. 1998). As stated before, $TGF-\upbeta$ has a global anabolic effect as it upregulates osteoclast apoptosis and increases the pool of osteoblast precursors. In this work, we have shown that considering this factor alone is insufficient to explain the bone loss due to ageing. We must admit that the decrease in $TGF-\upbeta$ had to be limited, establishing a minimum for $\alpha _{\text{TGF}-\upbeta }$, since otherwise the loss of bone mass estimated for women in their eighties was so high that the bone practically disappeared after that age. This limitation of the minimum of $\alpha _{\text{TGF}-\upbeta }$ is also justified by a recent work (Calvo-Gallego et al. 2023) in which we have shown that $TGF-\upbeta$ plays a fundamental role in the coordination of the sequence resorption-reversion-formation of the BMU and that if the concentration of $TGF-\upbeta$ is not sufficient, those three phases can become uncoordinated, something that, to our knowledge, has not been reported in elderly people.

The effect of changes in sclerostin production on ageing has not been considered to date in models of bone remodelling, although the increase in serum sclerostin levels with age had been shown in several clinical studies (Ardawi et al. 2011; Sharma-Ghimire et al. 2022). In contrast, the increase in sclerostin levels had been attributed to menopause and had therefore been included in the PMO simulations (Martin et al. 2019). In this paper, we have further analysed the role of sclerostin production and came to the conclusion that it should rather be considered to account for the effect of ageing, as suggested by clinical evidence. By doing so, our model was able to predict most of the bone loss that occurs with age. Nevertheless, the joint consideration of both factors (decrease in the concentration of $TGF-\upbeta$ and increase in the levels of sclerostin) slightly improves the accuracy of the prediction and would also be justified by clinical evidence. For those reason, it has been assumed as the best option to simulate the effect of ageing.

The simulations of ARO+PMO were carried out by adding the effect of oestrogen deficiency to that of ageing. Shevde et al. (2000) suggested that oestrogen modulates osteoclast formation both by down-regulating the expression of osteoclastogenic cytokines from supportive cells and by directly suppressing RANKL-induced osteoclast differentiation. Therefore, oestrogen deficiency will reverse this effect. More precisely, oestrogen deficiency produces an enhanced RANKL expression by stromal cells and lymphocytes (Eghbali-Fatourechi et al. 2003) and increases the responsiveness of osteoclasts to RANKL (Shevde et al. 2000) and those effects have been taken into account here through a production of RANKL external to bone ($P_{\text{RANKL}}^{\text{ED}}$) and a decrease in the constant $K_{\text{act,Oc}_\text{p}}^{\text{RANKL}}$, respectively. The third way to simulate oestrogen deficiency consisted in decreasing the secretion of OPG in osteoblasts (through $\beta_{\text {OPG,Ob}_\text{a}}$), which is enhanced by oestrogen receptor agonists in a normal state (Hofbauer et al. 1999; Viereck et al. 2003).

As shown in Fig. 3 and Table 2 for simulations S4 to S6, the three ways of modelling oestrogen deficiency yield similar fits of the average density values given by Riggs et al. (2004) in women. Even the combination of the three factors (simulation S7) does not provide a significant improvement. Therefore, it can be concluded that the three ways of modelling oestrogen deficiency have similar effects and any of them separately can be considered valid. The results discussed below are those obtained with the S4 simulation, that models oestrogen deficiency solely through an external production of RANKL, as done in previous works (Scheiner et al. 2014; Martínez-Reina and Pivonka 2019; Martínez-Reina et al. 2021a, b; Calvo-Gallego et al. 2022).

With the model proposed here, an accurate fit of the data obtained by Riggs et al. has been achieved for both men and women (see Figs. 2 and 3, respectively). Moreover, if only the effect of oestrogen deficiency is considered, we obtain a bone loss that stabilises over time (black line in Fig. 4), as suggested by Nordin et al. (1990) when these authors decomposed the effects of age and YSM and assigned the latter a logarithmic dependence. For the approximately linear bone loss with age, our results also agree with those of Nordin et al. It must be noted that the S-shape of bone loss curves in women (Fig. 3) is due to the following: the decrease in oestrogen just after menopause has a more marked effect in the short term, which attenuates later, about 10 years after menopause, as confirmed by Nordin et al. (1990) and the black lines in Fig. 4. After this stabilisation of bone loss, around the age of seventy, the ageing effect is more pronounced and bone loss accelerates again.

Notwithstanding the above, the study of the uncoupled effects of ageing and oestrogen deficiency may lead to the erroneous conclusion that osteoporosis will occur independently of menopause and that oestrogen deficiency has an effect on bone loss, but only to a limited extent. Certainly, such a conclusion can be drawn as the bone loss due to age alone is almost linear and the loss due to oestrogen deficiency alone stops after about 10 years. However, when simulating ARO+PMO, a certain coupling between the two effects was observed and the bone loss due to the joint effect has a steeper slope than when only the effect of age was considered after those 10 years, when the effect of oestrogen deficiency should supposedly have disappeared. In other words, oestrogen deficiency aggravates the bone loss that occurs naturally with ageing, and thus, in the ARO+PMO simulations (blue curve in Fig. 4), the bone loss is greater than the sum of the bone losses obtained when the effects are simulated separately and added together (green curve in Fig. 4).

In fact, if the effect of oestrogen deficiency stops over time, one might wonder what is the point of prescribing selective oestrogen receptor modulators (SERMs) such as raloxifene, which are intended to counteract the drop in oestrogen levels, a posteriori. In view of our results, the convenience of SERMs could be explained precisely by the need to combat the coupling that seems to exist between oestrogen deficiency and ageing. But then the question is whether this effect can be counteracted a posteriori or whether it would be better to tackle it from the beginning of the menopause as a prophylactic measure.

The variation of sclerostin levels with age is a controversial issue. There is a general consensus that serum levels are positively correlated with age Ardawi et al. (2011); Sharma-Ghimire et al. (2022), but this may contradict other ideas, as discussed below. Most authors agree that sclerostin is produced almost exclusively by osteocytes (Ardawi et al. 2011; Baron and Rawadi 2007; van Bezooijen et al. 2004). If the density of osteocytes in bone matrix is constant as assumed by Martin et al. (2019), the concentration of these cells would be proportional to $f_{bm}$ and, therefore, $f_{bm}$ would be proportional to the concentration of sclerostin, something that agrees with the positive correlation found by Sharma-Ghimire et al. (2022) between BMD and serum sclerostin. However, that would be in contradiction with the positive correlation between sclerostin levels and age, since $f_{bm}$ decreases with age. Even more so when the concentration of osteocytes in the bone matrix decreases markedly in senescence due to the increasing rate of osteocyte apoptosis, which leaves many empty lacunae and leads to the phenomenon known as micropetrosis (Milovanovic and Busse 2020). It is conceivable then that serum levels of sclerostin could not increase with age (or even remain constant) if it were only produced by osteocytes. Either there are other sources of production, as suggested by several authors (Kusu et al. 2003; Pederson et al. 2009; Ota et al. 2013; Zhu et al. 2011; Ke et al. 2012), or its clearance rate decreases with age Roforth et al. (2014). In this work, we have opted for the former, that is, to assume an external supply of sclerostin to the bone.

According to the results of our simulations, the production of sclerostin within bone, which would correspond to the production due to osteocytes (red line Fig. 5 left), decreases after menopause, which could be explained by the lower osteocyte population resulting from the lower $f_{bm}$ and is in agreement with the studies of Jastrzebski et al. (2013). These authors measured sclerostin mRNA levels in different bones of ovariectomized mice (OVX) and compared that production index with that of sham-operated mice (SHAM), detecting a significant decrease in sclerostin mRNA levels of about 50% after ovariectomy compared to SHAM. However, they did not detect significant differences in serum sclerostin levels between both groups. Although with our model it is not possible to assess serum sclerostin, but only the concentration of sclerostin in bone, it is reasonable to think that both may be related and we did not find a significant fall in the latter after menopause, but only a slight decrease.

We analysed in Fig. 6 the evolution of bone turnover rate over time. Our model is only able to evaluate local but not systemic variables such as serum concentrations. Thus, it cannot be used to assess bone turnover markers (BTM) quantitatively. However, our model is able to evaluate changes in local BTM such as bone resorption and formation rates and these changes can be extrapolated to the systemic level to allow at least a quantitative comparison. The dashed lines correspond to men, for whom only the effect of age has been simulated. The evolution is congruent with that observed by Fatayerji and Eastell (1999), who found the highest BTMs in young people, the lowest in the fifth and sixth decade and a small increase in some bone formation markers in the eighth decade. The solid lines correspond to women for whom the effect of oestrogen deficiency has been added to that of ageing, from the age of 49 onwards. The former greatly increases the resorption rate and to a lesser extent the formation rate, at least in the years immediately following the drop in oestrogen, since the formation rate does increase notably in the mid-term. These results are qualitatively consistent with the findings of Ebeling et al. (1996) who observed an increase in resorption BTMs already in the perimenopause and accentuated in the postmenopause, but did not observe significant changes in formation markers until the postmenopause.

The analysis of the influence of age at menopause on bone mass loss (Fig. 7) showed that early menopause can be dangerous as it significantly reduces average density from an early age and keeps the risk of fracture high for a larger part of the patient’s life. In the long term, the BDL curves converge and the subsequent bone mass loss would be explained only by the ageing effect. This result reinforces the idea that oestrogen deficiency has only a temporal effect, as shown before in Fig. 4 (see black lines) and suggested by Nordin et al. (1990).

The variability of the age at menopause and the high influence it has on short-term BDL could explain the high dispersion of the density results obtained by Riggs et al. (2004) in women between 50 and 60 years of age. Another possible source of dispersion is the variability of the subject’s level of physical activity, which has not been taken into account in this work, though it can have a major impact on BDL, especially in older people.

The simulation of the treatment with Dmab highlighted the importance of considering not only YSM but also age in the effectiveness of the treatment. This feature is a novelty of the present work compared to previous studies in which we were only able to analyse the effect of YSM and an important step forward in the search for a patient-specific treatment. The results showed that a gap of 10 years between menopause and the start of treatment results in a similar BDG regardless of the age at menopause (see Fig. 8 left). However, a young patient, with early menopause but no other complications, might not yet need treatment if her density is not so low as to compromise bone integrity. On the other hand, the treatment does not seem as effective if YSM<10 years (see Fig. 8 right, blue curve) as BDG is lower in absolute terms and treatment is probably not necessary either. It can therefore be concluded that under normal circumstances and as long as there are no other factors that may have aggravated the disease, it would not be justified to start treatment earlier than 10 years after menopause or before the age of 60.

Only the influence of age and YSM on the efficacy of Dmab treatment is discussed here, but obviously the decision to start treatment is determined mainly by the risk of fracture and this, in turn, may be influenced by other factors such as physical condition, family history of osteoporotic fractures, and the concurrence of other pathologies.

We must mention some limitations of our study. We have simulated, respectively, two and three mechanisms to consider the effect of ageing and menopause on bone turnover, but both processes are very complex and may involve further mechanisms not considered here, which need to be investigated in the future. One of them, and a very relevant one, is the alteration of the immune system throughout the life cycle. It has been reported that a variety of cytokines and growth factors produced by immune cells influence the function of bone cells (Lorenzo 2000) and should be considered in BCPM. Therefore, indirect effects of altered immune status in postmenopausal women might contribute to ongoing bone destruction (Fischer and Haffner-Luntzer 2022). Also, the effects of ageing on the immune system are manifested at multiple levels that include reduced production of B and T cells in bone marrow, which might alter the production of cytokines end eventually the functioning of bone cells (Montecino-Rodriguez et al. 2013). Cellular senescence may also have an important effect on bone turnover. Some authors have shown that senescence-associated secretory phenotype (SASP) factors promote survival of osteoclast progenitors, inhibit osteoblasts differentiation (Farr et al. 2017) and alter lineage commitment of mesenchymal stem cells towards the adipocyte and away from the osteoblast lineage (Farr and Khosla 2019), but none of these effects were specifically considered in our model. Finally, we have assumed a constant density of osteocytes within bone matrix, $\eta$, (see Eq. (5)), but this would not valid in senescence, when the increasing rate of osteocyte apoptosis leaves many empty lacunae (Milovanovic and Busse 2020).

## Summary and conclusions

A previously developed BCPM was adapted in this work to consider the effect of ageing and the oestrogen decay. It was used to simulate the bone loss produced by ageing and menopause. The following conclusions could be drawn from the simulations:

- Ageing leads to a decreasing content of TGF-$\upbeta$ in bone matrix and to an increasing production of sclerostin by non-skeletal cells, but the effect of the latter on bone loss is more important.
- Oestrogen deficiency has been reported to increase RANKL expression, decrease OPG production and increase the responsiveness of osteoclasts to RANKL, but the three effects have similar consequences when considered in our model.
- The effects of ageing and oestrogen deficiency could be coupled, as the concurrence of both manifests in a more pronounced disease state than the mere superimposition of the bone losses simulated for each factor separately.
- Our model predicts a significant decrease in the average BMD for women with an early menopause already in their fifties, though in their sixties the simulated bone loss is independent of the age at menopause.
- The efficacy of denosumab seems to depend on the years elapsed since menopause, so that commencing the treatment would not be justified earlier than 10 years after menopause but either in women younger than 60 years, if bone mass has not yet been significantly reduced.

## Supplementary Information

Below is the link to the electronic supplementary material.

Supplementary file1 (PDF 359 KB)

## Abbreviations

ARO: Age-related osteoporosis
BCPM: Bone cell population model
BDL: Bone density loss
BDG: Bone density gain
BMD: Bone mineral density
BMU: Basic multicellular unit
OP: Osteoporosis
OPG: Osteoprotegerin
PMO: Postmenopausal osteoporosis
PTH: Parathyroid hormone
RANK: Receptor activator of nuclear factor $\kappa \upbeta$
RANKL: Receptor activator of nuclear factor $\kappa \upbeta$ ligand
RMSE: Root mean square error
SED: Strain energy density
SERM: Selective oestrogen receptor modulator
TGF-$\upbeta$: Transforming growth factor $\upbeta$
TSM: Time since menopause (in days)
TSSM: Time since skeletal maturity (in days)
vBMD: Volumetric bone mineral density
YSM: Years since menopause
