# Supplementary material for: An in silico approach to elucidate the pathways leading to primary osteoporosis: age-related vs. postmenopausal
Source: Biomech Model Mechanobiol. 2024 May 3;23(4):1393–409. doi: 10.1007/s10237-024-01846-2 (PMC11584493; doi:10.1007/s10237-024-01846-2)
Supplement: Supplementary file 1 — Supplementary file1 (PDF 359 KB) [file 10237_2024_1846_MOESM1_ESM.pdf]

Supplementary Material to: An in-silico approach to elucidate the pathways leading to primary osteoporosis: age-related vs. postmenopausal

Rocío Ruiz-Lozano[a,1], José Luis Calvo-Gallego[a,1], Peter Pivonka[b,1], Michelle McDonald[c,1], Javier Martínez-Reina[a,1,*]

[a] *Departmento de Ingeniería Mecánica y Fabricación, Universidad de Sevilla, Seville 41092, Spain*
[b] *School of Mechanical, Medical and Process Engineering, Queensland University of Technology, QLD 4000, Australia*
[c] *Garvan Institute of Medical Research, Sydney, Australia*

1. Introduction

In this document we provide those details of the mathematical model not provided in the main document. More precisely, we explain here those features of the original model by Martin et al. [1] which remained unchanged; while those that have been modified or are more relevant to the study are presented in the main document.

This document is structured as follows: In section 2 we present the general equations for competitive binding between ligands and receptors; the specific equations for the competitive binding of the complex Wnt–Scl–LRP5/6 are given in section 3 and the co-regulation of RANKL levels via the antagonistic effect of PTH and nitric oxide in section 4. The equations for the competitive binding of the complex RANK-RANKL-OPG-Dmab were given in the main document. The upregulation of RANKL expressed by osteocytes due to damage is given in section 5. The equations related to the regulatory effect of TGF-β are given in section 7. Section 8 describes the term of proliferation of osteoblast precursors. Finally, the model constants are given in Table 2.

*Corresponding author

2. Competitive binding

Many biological processes are controlled by binding of biochemical factors which act as receptor and ligand. In some of them two or more ligands compete to bind to the receptor. This is the case of Wnt and sclerostin that compete to bind to LRP5/6 to control the proliferation of osteoblast precursors and also the case of RANK, OPG and Dmab which compete to bind to RANKL to control the differentiation of osteoclast precursors into mature active osteoclasts.

Let us consider separately the binding of a given receptor R to its ligands A and B to form, respectively, the complexes A–R and B–R. Let us consider for each species X=A,B,R a production term P_{X} and a degradation term D_{X}, along with a degradation term for the complex $D_{\mathrm{X-Y}}$. Let $K^{r}_{\mathrm{X-Y}}$ and $K^{f}_{\mathrm{X-Y}}$ be the reverse and forward binding reaction constants, respectively.

$$\begin{array}{ccccccc} \downarrow P_{\mathrm{R}} & & \downarrow P_{\mathrm{A}} & & & & \\ \mathrm{R} & + & \mathrm{A} & \underset{K^{r}_{\mathrm{A-R}}}{\overset{K^{f}_{\mathrm{A-R}}}{\rightleftharpoons}} & \mathrm{A-R} & \overset{D_{\mathrm{A-R}}}{\rightharpoonup} & \emptyset \\ \downarrow D_{\mathrm{R}} & & \downarrow D_{\mathrm{A}} & & & & \end{array} \tag{1}$$

$$\begin{array}{ccccccc} \downarrow P_{\mathrm{R}} & & \downarrow P_{\mathrm{B}} & & & & \\ \mathrm{R} & + & \mathrm{B} & \underset{K^{r}_{\mathrm{B-R}}}{\overset{K^{f}_{\mathrm{B-R}}}{\rightleftharpoons}} & \mathrm{B-R} & \overset{D_{\mathrm{B-R}}}{\rightharpoonup} & \emptyset \\ \downarrow D_{\mathrm{R}} & & \downarrow D_{\mathrm{B}} & & & & \end{array} \tag{2}$$

The law of mass action provides the following set of differential equations:

$$\frac{\partial[\mathrm{A-R}]}{\partial t} = K^{f}_{\mathrm{A-R}}\,[\mathrm{A}]\,[\mathrm{R}] - K^{r}_{\mathrm{A-R}}\,[\mathrm{A-R}] - \tilde{D}_{\mathrm{A-R}}\,[\mathrm{A-R}] \tag{3a}$$

$$\frac{\partial[\mathrm{B-R}]}{\partial t} = K^{f}_{\mathrm{B-R}}\,[\mathrm{B}]\,[\mathrm{R}] - K^{r}_{\mathrm{B-R}}\,[\mathrm{B-R}] - \tilde{D}_{\mathrm{B-R}}\,[\mathrm{B-R}] \tag{3b}$$

$$\frac{\partial[\mathrm{A}]}{\partial t} = P_{\mathrm{A}} - \tilde{D}_{\mathrm{A}}\,[\mathrm{A}] + K^{r}_{\mathrm{A-R}}\,[\mathrm{A-R}] - K^{f}_{\mathrm{A-R}}\,[\mathrm{A}]\,[\mathrm{R}] \tag{3c}$$

$$\frac{\partial[\mathrm{B}]}{\partial t} = P_{\mathrm{B}} - \tilde{D}_{\mathrm{B}}\,[\mathrm{B}] + K^{r}_{\mathrm{B-R}}\,[\mathrm{B-R}] - K^{f}_{\mathrm{B-R}}\,[\mathrm{B}]\,[\mathrm{R}] \tag{3d}$$

$$\begin{aligned}\frac{\partial[\mathrm{R}]}{\partial t} =& P_{\mathrm{R}} - \tilde{D}_{\mathrm{R}}\,[\mathrm{R}] + K^{r}_{\mathrm{A-R}}\,[\mathrm{A-R}] + K^{r}_{\mathrm{B-R}}\,[\mathrm{B-R}] \\ &- K^{f}_{\mathrm{A-R}}\,[\mathrm{A}]\,[\mathrm{R}] - K^{f}_{\mathrm{B-R}}\,[\mathrm{B}]\,[\mathrm{R}]\end{aligned} \tag{3e}$$

where $[\mathrm{X}]$ and $[\mathrm{X-Y}]$ represent, respectively, the concentration of species X and complex X-Y. The degradation terms are assumed proportional to the concentration of the species, i.e. $D_{\mathrm{X}} = \tilde{D}_{\mathrm{X}}\,[\mathrm{X}]$, with $\tilde{D}_{\mathrm{X}}$ being the degradation rate.

Following Pivonka et al. [2] we assume that the binding reactions are much faster than the cell responses they produce and hence a quasi-steady state can be assumed, implying that the time derivatives of Eqs. (3) are null. This condition in Eqs. (3a) and (3b) yield:

$$[\mathrm{A-R}] = \frac{[\mathrm{A}]\,[\mathrm{R}]}{K_{\mathrm{A-R}}} \tag{4a}$$

$$[\mathrm{B-R}] = \frac{[\mathrm{B}]\,[\mathrm{R}]}{K_{\mathrm{B-R}}} \tag{4b}$$

where:

$$K_{\mathrm{A-R}} = \frac{K^{r}_{\mathrm{A-R}} + \tilde{D}_{\mathrm{A-R}}}{K^{f}_{\mathrm{A-R}}} \tag{5a}$$

$$K_{\mathrm{B-R}} = \frac{K^{r}_{\mathrm{B-R}} + \tilde{D}_{\mathrm{B-R}}}{K^{f}_{\mathrm{B-R}}} \tag{5b}$$

The stationarity condition of Eqs. (3c)-(3e) yields:

$$[\mathrm{A}] = \frac{P_{\mathrm{A}}}{\tilde{D}_{\mathrm{A}} + \frac{\tilde{D}_{\mathrm{A-R}}}{K_{\mathrm{A-R}}}\,[\mathrm{R}]} \tag{6a}$$

$$[\mathrm{B}] = \frac{P_{\mathrm{B}}}{\tilde{D}_{\mathrm{B}} + \frac{\tilde{D}_{\mathrm{B-R}}}{K_{\mathrm{B-R}}}\,[\mathrm{R}]} \tag{6b}$$

$$[\mathrm{R}] = \frac{P_{\mathrm{R}}}{\tilde{D}_{\mathrm{R}} + \frac{\tilde{D}_{\mathrm{A-R}}}{K_{\mathrm{A-R}}}\,[\mathrm{A}] + \frac{\tilde{D}_{\mathrm{B-R}}}{K_{\mathrm{B-R}}}\,[\mathrm{B}]} \tag{6c}$$

If the degradation ($\tilde{D}_{\mathrm{X}}$,$\tilde{D}_{\mathrm{X-Y}}$), production (P_{X}) and dissociation constants, ($K_{\mathrm{X-Y}}$) are known, (6) constitutes a non-linear system of three equations with three unknowns, namely [A], [B], [R].

The total concentration of a receptor is the sum of the concentrations of the receptor which is found free and bound to ligands, i.e.:

$$[\mathrm{R}]_{\mathrm{tot}} = [\mathrm{R}] + [\mathrm{A-R}] + [\mathrm{B-R}] = [\mathrm{R}]\left(1 + \frac{[\mathrm{A}]}{K_{\mathrm{A-R}}} + \frac{[\mathrm{B}]}{K_{\mathrm{B-R}}}\right) \tag{7}$$

where Eqs. (4) have been used. The stationarity condition is equivalent to establish that the production rate of a species must equal the degradation rate, including all its forms, free and bound. For instance, in the case of the receptor that can bind to different ligands, this condition reads:

$$P_{\mathrm{R}} = \tilde{D}_{\mathrm{R}}\,[\mathrm{R}] + \sum_{\mathrm{L}} \tilde{D}_{\mathrm{L-R}}\,[\mathrm{L-R}] \tag{8}$$

which can be obtained from Eqs. (3a), (3b) and (3e) by imposing that the stationarity condition is met, ($\frac{\partial[\mathrm{R}]}{\partial t} = \frac{\partial[\mathrm{L-R}]}{\partial t} = 0 \quad \forall\, \mathrm{L} = \mathrm{A}, \mathrm{B}$). In the case of a ligand, that only binds to the receptor, that condition reads:

$$P_{\mathrm{L}} = \tilde{D}_{\mathrm{L}}\,[\mathrm{L}] + \tilde{D}_{\mathrm{L-R}}\,[\mathrm{L-R}] \tag{9}$$

The degradation rates are usually assumed as constants but the production rates are modelled in a more complex way. For instance, the production rate of ligand L can be split into a term corresponding to endogenous production, $P_{\mathrm{L,b}}$, and a term accounting for external dosage, $P_{\mathrm{L,d}}$:

$$P_{\mathrm{L}} = P_{\mathrm{L,b}} + P_{\mathrm{L,d}} \tag{10}$$

The endogenous production is sometimes modelled by the following equation:

$$P_{\mathrm{L,b}} = \sum_{\mathrm{X,Y}} \beta_{\mathrm{L,Y}}\, \pi^{\mathrm{X}}_{\mathrm{act/rep,Y}}\, \mathrm{Y}\left(1 - \frac{[\mathrm{L}]}{[\mathrm{L}]_{\mathrm{max}}}\right) \tag{11}$$

where Y is the concentration of the cell type Y producing L with a production rate $\beta_{\mathrm{L,Y}}$, regulated by the species X through the activator or repressor

function $\pi^{X}_{act/rep,Y}$. The parenthesis establishes a saturation condition in such a way that ligand is not produced if its concentration reaches the maximum or saturation value, $[L]_{max}$.

As described by Pivonka et al. [3] the activation of a certain biological process regulated by the formation of the complex L–R is given by the ratio between the receptors R occupied by ligands L and the total number of receptors:

$$\pi^{L}_{act,Y} = \frac{[L-R]}{[R]_{tot}} = \frac{[L-R]}{[R] + \sum_{L'} [L'-R]} \tag{12}$$

Similarly, the repressor action of the binding is given by the complementary to one of the latter:

$$\pi^{L}_{rep,Y} = \frac{[R]_{tot} - [L-R]}{[R]_{tot}} = \frac{[R] + \sum_{L' \neq L} [L'-R]}{[R] + \sum_{L'} [L'-R]} \tag{13}$$

In case of a single ligand the latter expressions yield the first-order Hill activator and repressor functions:

$$\pi^{L}_{act,Y} = \frac{[L]}{[L] + K^{L-R}_{act,Y}} \tag{14}$$

$$\pi^{L}_{rep,Y} = \frac{K^{L-R}_{rep,Y}}{K^{L-R}_{rep,Y} + [L]} \tag{15}$$

3. Competitive binding Wnt–Scl–LRP5/6

The previous equations can be used to describe the competitive binding Wnt–Scl–LRP5/6. Wnt signaling is an anabolic pathway promoting the proliferation of osteoblast precursors and hence bone formation. Extracellular Wnt binds to Frizzled and the lipoprotein receptor-related protein LRP5/6, so triggering intracellular activation of β-catenin. Sclerostin, produced by osteocytes, modulates the signaling pathway by its interaction

with LRP5/6 receptors. This prevents the formation of the Wnt-Frizzled-LRP5/6 complex and therefore hinders preosteoblasts proliferation. Competitive Wnt–Scl–LRP5/6 binding is modelled as follows. First, Eq. (7) reads for LRP5/6:

$$[\mathrm{LRP5/6}]_{\mathrm{tot}} = [\mathrm{LRP5/6}] \cdot \left(1 + \frac{[\mathrm{Wnt}]}{K_{\mathrm{Wnt-LRP5/6}}} + \frac{[\mathrm{Scl}]}{K_{\mathrm{Scl-LRP5/6}}}\right) \tag{16}$$

The production of sclerostin is given by an equation like (9), which now reads:

$$P_{\mathrm{Scl,b}} + P_{\mathrm{Scl,d}} = \tilde{D}_{\mathrm{Scl}}\,[\mathrm{Scl}] + \tilde{D}_{\mathrm{Scl-LRP5/6}}\,[\mathrm{Scl-LRP5/6}] \tag{17}$$

where $\tilde{D}_{\mathrm{Scl}}$ and $\tilde{D}_{\mathrm{Scl-LRP5/6}}$ are the degradation rates of sclerostin and the sclerostin-LRP5/6 complex, respectively. The concentration of this complex is given by the receptor-ligand binding equation (3a), which reads here:

$$[\mathrm{Scl-LRP5/6}] = \frac{[\mathrm{Scl}]\,[\mathrm{LRP5/6}]}{K_{\mathrm{Scl-LRP5/6}}} \tag{18}$$

The external dosage of sclerostin, $P_{\mathrm{Scl,d}}$, is set to zero and the endogenous production of sclerostin by osteocytes is:

$$P_{\mathrm{Scl,b}} = \beta_{\mathrm{Scl,Ot}}\,\pi^{\Psi_{\mathrm{bm}}}_{\mathrm{rep,Scl}}\,\mathrm{Ot}\left(1 - \frac{[\mathrm{Scl}]}{[\mathrm{Scl}]_{\mathrm{max}}}\right) \tag{19}$$

where $\beta_{Scl,Ot}$ and $[\mathrm{Scl}]_{\mathrm{max}}$ are, respectively, the sclerostin production rate and its maximum concentration. The production of sclerostin by osteocytes is downregulated by the mechanical stimulus through the repressor function $\pi^{\Psi_{\mathrm{bm}}}_{\mathrm{rep,Scl}}$ (see Eq. (35) later on). Replacing (19) and (18) into (17) yields:

$$\beta_{\mathrm{Scl,Ot}}\,\pi^{\Psi_{\mathrm{bm}}}_{\mathrm{rep,Scl}}\,\mathrm{Ot}\left(1 - \frac{[\mathrm{Scl}]}{[\mathrm{Scl}]_{\mathrm{max}}}\right) = \tilde{D}_{\mathrm{Scl}}\,[\mathrm{Scl}] + \tilde{D}_{\mathrm{Scl-LRP5/6}}\,\frac{[\mathrm{Scl}]\,[\mathrm{LRP5/6}]}{K_{\mathrm{Scl-LRP5/6}}} \tag{20}$$

Following Martin et al. [1] we assumed that the total number of LRP5/6 receptors per osteoblast precursor ($N^{\mathrm{LRP5/6}}_{\mathrm{OB_p}}$) is constant and thus:

$$[\mathrm{LRP5/6}]_{\mathrm{tot}} = N_{\mathrm{OB_p}}^{\mathrm{LRP5/6}}\,\mathrm{Ob_p} \tag{21}$$

Solving for [LRP5/6] in Eq. (16) and replacing it into (20) yields the following second-order polynomial of the free sclerostin, [Scl]:

$$A\,[\mathrm{Scl}]^2 + B\,[\mathrm{Scl}] + C = 0 \tag{22}$$

where the constants coefficients of the polynomial are:

$$A = \tilde{D}_{\mathrm{Scl}} + \frac{\beta_{\mathrm{Scl,Ot}}\,\pi_{\mathrm{rep,Scl}}^{\Psi_{\mathrm{bm}}}\,\mathrm{Ot}}{[\mathrm{Scl}]_{\mathrm{max}}} \tag{23}$$

$$B = A\cdot K_{\mathrm{Scl-LRP5/6}}\left(1+\frac{[\mathrm{Wnt}]}{K_{\mathrm{Wnt-LRP5/6}}}\right) + \tilde{D}_{\mathrm{Scl-LRP5/6}}\,[\mathrm{LRP5/6}]_{\mathrm{tot}} - \left(P_{\mathrm{Scl,d}} + \beta_{\mathrm{Scl,Ot}}\,\pi_{\mathrm{rep,Scl}}^{\Psi_{\mathrm{bm}}}\,\mathrm{Ot}\right) \tag{24}$$

$$C = -K_{\mathrm{Scl-LRP5/6}}\left(1+\frac{[\mathrm{Wnt}]}{K_{\mathrm{Wnt-LRP5/6}}}\right)\left(P_{\mathrm{Scl,d}} + \beta_{\mathrm{Scl,Ot}}\,\pi_{\mathrm{rep,Scl}}^{\Psi_{\mathrm{bm}}}\,\mathrm{Ot}\right) \tag{25}$$

$$\tag{26}$$

We can use (21) in the previous expression together with the previously calculated cell populations and [Wnt], which is assumed constant, to work out the three coefficients. Only one solution of (22) is positive as shown by Martin et al. [1] and this solution [Scl] is then used in (16) to calculate [LRP5/6]. Finally, using Eqs. (12) and (21), the activator function in the Ob_p proliferation term can be calculated as:

$$\pi_{\mathrm{act,Ob_p}}^{\mathrm{Wnt}} = \frac{[\mathrm{Wnt-LRP5/6}]}{[\mathrm{LRP5/6}]_{\mathrm{tot}}} = \frac{[\mathrm{Wnt}]\,[\mathrm{LRP5/6}]}{K_{\mathrm{Wnt-LRP5/6}}\,[\mathrm{LRP5/6}]_{\mathrm{tot}}} \tag{27}$$

4. Co-regulation of RANKL via PTH and NO concentration

RANKL transcription is upregulated by parathyroid hormone (PTH) and downregulated by nitric oxide (NO). In the model developed by Martin et al.

[1] this antagonistic influence was merged into a co-regulatory function capturing both effects.

$$\pi^{\text{PTH,NO}}_{\text{act/rep,RANKL}} = \lambda_s \left(\pi^{\text{PTH}}_{\text{act,RANKL}} + \pi^{\text{NO}}_{\text{rep,RANKL}}\right) + \lambda_c \, \pi^{\text{PTH}}_{\text{act,RANKL}} \cdot \pi^{\text{NO}}_{\text{rep,RANKL}} \tag{28}$$

where the activator function accounting for the effect of PTH is:

$$\pi^{\text{PTH}}_{\text{act,RANKL}} = \frac{[\text{PTH}]}{[\text{PTH}] + K^{\text{PTH}}_{\text{act}}} \tag{29}$$

and the repressor effect on OPG (see Eq. (10) in the main document) is accounted for through the function:

$$\pi^{\text{PTH}}_{\text{rep,Ob}_\text{a}} = \frac{K^{\text{PTH}}_{\text{rep}}}{[\text{PTH}] + K^{\text{PTH}}_{\text{rep}}} \tag{30}$$

being $K^{\text{PTH}}_{\text{act}}$ and $K^{\text{PTH}}_{\text{rep}}$ constants and the concentration of PTH given by:

$$[\text{PTH}] = \frac{\beta_{\text{PTH}}}{\tilde{D}_{\text{PTH}}} \tag{31}$$

which comes from Eqs. (8) to (11) when there is no ligand for the species, the external dosage is null ($P_{\text{PTH,d}} = 0$), the endogenous production rate is not regulated ($\pi^{\text{X}}_{\text{act/rep,Y}} \cdot \text{Y} = 1$ see Eq. (11)) and the saturation value $[\text{PTH}]_{\max}$ is large enough to assume the parenthesis equal to 1. β_{PTH} and $\tilde{D}_{\text{PTH}}$ are the endogenous production and degradation rate of PTH, respectively. On the other hand, the factor corresponding to nitric oxide is:

$$\pi^{\text{NO}}_{\text{rep,RANKL}} = \frac{K^{\text{NO}}_{\text{rep}}}{[\text{NO}] + K^{\text{NO}}_{\text{rep}}} \tag{32}$$

with $K^{\text{NO}}_{\text{rep}}$ a constant and the concentration of NO given by:

$$[\text{NO}] = \frac{P_{\text{NO,d}} + \beta_{\text{NO,Ot}} \, \pi^{|\varepsilon|_{\max}}_{\text{act,NO}} \, \text{Ot}}{\tilde{D}_{\text{NO}} + \frac{\beta_{\text{NO,Ot}} \, \pi^{|\varepsilon|_{\max}}_{\text{act,NO}} \, \text{Ot}}{[\text{NO}]_{\max}}} \tag{33}$$

which also comes from Eq. (8) in the absence of ligands. The external dosage of nitric oxide $P_{\mathrm{NO,d}}$ is set to zero in this study, $\beta_{\mathrm{NO,Ot}}$, $\tilde{D}_{\mathrm{NO}}$ and $[\mathrm{NO}]_{\mathrm{max}}$ are, respectively, the endogenous production and degradation rate of nitric oxide and its maximum content. The factor $\pi_{\mathrm{act,NO}}^{\Psi_{\mathrm{bm}}}$ is the mechanical feedback activator function that accounts for the production of NO by osteocytes. This function and the repressor function affecting the production of sclerostin by osteocytes (see Eq. (19)) are defined by the following sigmoidal functions:

$$\pi_{\mathrm{act,NO}}^{|\varepsilon|_{\mathrm{max}}} = \rho_{\mathrm{act}} + \frac{(\alpha_{\mathrm{act}} - \rho_{\mathrm{act}})\ |\varepsilon|_{\mathrm{max}}}{\delta_{\mathrm{act}}^{\gamma^{\mathrm{act}}} + |\varepsilon|_{\mathrm{max}}^{\gamma^{\mathrm{act}}}} \tag{34}$$

$$\pi_{\mathrm{rep,Scl}}^{|\varepsilon|_{\mathrm{max}}} = \alpha_{\mathrm{rep}} - \frac{(\alpha_{\mathrm{rep}} - \rho_{\mathrm{rep}})\ |\varepsilon|_{\mathrm{max}}^{\gamma^{\mathrm{rep}}}}{\delta_{\mathrm{rep}}^{\gamma^{\mathrm{rep}}} + |\varepsilon|_{\mathrm{max}}^{\gamma^{\mathrm{rep}}}} \tag{35}$$

where $\rho_{\sim}$ and $\alpha_{\sim}$ are, respectively, the minimum and maximum anticipated response, $\gamma^{\sim}$ is the sigmoidicity, influencing the steepness of the response, $\delta_{\sim}$ is the value of the stimulus producing the half-maximal response [4], and $|\varepsilon|_{max}$ is the maximun principal strain in absolute value.

5. Upregulation of RANKL expressed by osteocytes due to microstructural damage

As proposed in [5] we have assumed that RANKL expression by osteocytes is upregulated by the presence of microstructural damage in the bone matrix through the factor $\pi_{\mathrm{act,RANKL}}^{\mathrm{dam}}$, which is defined as a sigmoidal function of damage, d:

$$\pi_{\mathrm{act,RANKL}}^{\mathrm{dam}} = \rho_{dam} + (\alpha_{dam} - \rho_{dam})\ (1 + K_{dam})\ \frac{d}{K_{dam} + d} \tag{36}$$

where K_{dam} is a constant, ρ_{dam} is the minimum value of the factor $\pi_{\mathrm{act,RANKL}}^{\mathrm{dam}}$, corresponding to $d = 0$, while α_{dam} is its maximum value, corresponding to $d = 1$.

6. Two-Compartment PK Model of Denosumab

Following the pharmacokinetic model developed by Martínez-Reina et al. [6], a first-order rate process (k_a) governs the absorption of the drug ($Dose$)

from the subcutaneous (SC) injection site into the central compartment ($[Dmab]_{CC}$), being V_c/F the volume of the central compartment adjusted for bioavailability. The drug elimination from the central compartment is described by a combination of a linear first-order process (k_{el}) and a non-linear saturation process (V_{max}, K_m):

$$\frac{d\,[Dmab]_{CC}}{dt} = \frac{Dose}{V_c/F} k_a e^{-k_a t} - \left[k_{el}\,[Dmab]_{CC} + \frac{V_{max}}{V_c/F} \frac{[Dmab]_{CC}}{K_m + [Dmab]_{CC}} \right] \quad (37)$$

In Eq. 37 *Dose* is given in ng per kg of body weight and then $[Dmab]_{CC}$ is calculated in ng/ml and subsequently converted into pmol/l, through the molecular weight of denosumab $M_{Dmab} = 149$ kDa.

In previous models (Martínez-Reina and Pivonka [7], Martínez-Reina et al. [8]) they have assumed that a fraction of the Dmab present in the central compartment was available in the bone compartment to compete with RANK to bind to RANKL. However, this availability actually implied a reversible exchange of Dmab between both compartments. Given the affinity of Dmab for RANKL, which is expressed by osteoblasts precursors within the bone compartment, a flux from the central compartment to the bone compartment seems more plausible than a reversible exchange. To this end, they added the bone compartment to the PK model in Martínez-Reina et al. [6]. The term in square brackets in Eq. 37 represents the elimination from the central compartment in the model of Marathe et al. [9] We have assumed that only a fraction $(1-\zeta)$ of this term is actually eliminated via urine and the rest, ζ, is the flux of denosumab into the bone compartment. In turn, the latter fraction can be considered as $P_{Dmab_{BC}}$, the production term of denosumab in the competitive binding reactions between RANKL, OPG and Dmab:

$$P_{Dmab_{BC}} = \zeta \left[k_{el}\,[Dmab]_{CC} + \frac{V_{max}}{V_c/F} \frac{[Dmab]_{CC}}{K_m + [Dmab]_{CC}} \right] \quad (38)$$

This production rate can be replaced in the expression that gives the concentration of ligands in competitive binding reactions (See Section 2), ie:

$$[Dmab]_{BC} = \frac{P_{Dmab_{BC}}}{\tilde{D}_{Dmab_{BC}} + \frac{\tilde{D}_{RANKL-Dmab}}{K_{RANKL-Dmab}} \cdot [RANKL]} \quad (39)$$

7. Regulatory role of TGF-β

TGF–β is stored in the bone matrix and released during resorption by osteoclasts. Its concentration is calculated following Pivonka et al. [2]:

$$[\mathrm{TGF}-\beta] = \frac{\alpha_{\mathrm{TGF}-\beta}\, k_{res}\, Oc_a}{\tilde{D}_{\mathrm{TGF}-\beta}} \tag{40}$$

where $\alpha_{\mathrm{TGF}-\beta}$ is the concentration of TGF–β in bone matrix and $\tilde{D}_{\mathrm{TGF}-\beta}$ is the TGF–β degradation rate. The concentration of TGF–β is used to define the activator/repressor functions in Eqs. (1)-(4) of the main document. These functions control the upregulation of the differentiation of $\mathrm{Ob_u}$ into $\mathrm{Ob_p}$, the upregulation of osteoclast apoptosis and the downregulation of the differentiation of $\mathrm{Ob_p}$ into $\mathrm{Ob_a}$:

$$\pi^{\mathrm{TGF}-\beta}_{\mathrm{act,Ob_u}} = \pi^{\mathrm{TGF}-\beta}_{\mathrm{act,Oc_p}} = \frac{[\mathrm{TGF}-\beta]}{K^{\mathrm{TGF}-\beta}_{\mathrm{act}} + [\mathrm{TGF}-\beta]} \tag{41}$$

$$\pi^{\mathrm{TGF}-\beta}_{\mathrm{rep,Ob_p}} = \frac{K^{\mathrm{TGF}-\beta}_{\mathrm{rep}}}{K^{\mathrm{TGF}-\beta}_{\mathrm{rep}} + [\mathrm{TGF}-\beta]} \tag{42}$$

with $K^{\mathrm{TGF}-\beta}_{\mathrm{act}}$ and $K^{\mathrm{TGF}-\beta}_{\mathrm{rep}}$ the activation and repression constants, respectively.

8. Proliferation of osteoblast precursors

Let us recall the differential equation of osteoblast precursors.

$$\begin{aligned}\frac{d\,\mathrm{Ob_p}}{dt} &= D_{\mathrm{Ob_u}} \cdot \mathrm{Ob_u} \cdot \pi^{\mathrm{TGF}-\beta}_{\mathrm{act,Ob_u}} - D_{\mathrm{Ob_p}} \cdot \mathrm{Ob_p} \cdot \pi^{\mathrm{TGF}-\beta}_{\mathrm{rep,Ob_p}} \\ &+ P_{\mathrm{Ob_p}} \cdot \mathrm{Ob_p} \cdot \pi^{\mathrm{Wnt}}_{\mathrm{act,Ob_p}}\end{aligned} \tag{43}$$

We can rewrite this equation as:

$$\frac{d\,\mathrm{Ob_p}}{dt} = \mathcal{D}_{\mathrm{Ob_u}} \cdot \mathrm{Ob_u} - \mathcal{D}_{\mathrm{Ob_p}} \cdot \mathrm{Ob_p} + \mathcal{P}_{\mathrm{Ob_p}} \cdot \mathrm{Ob_p} \tag{44}$$

where the terms in the right-hand side correspond, respectively, to the differentiation of $\mathrm{Ob_u}$ into $\mathrm{Ob_p}$, the differentiation of $\mathrm{Ob_p}$ into $\mathrm{Ob_a}$ and the proliferation of $\mathrm{Ob_p}$ at a rate $\mathcal{P}_{\mathrm{Ob_p}}$ which is determined by $P_{\mathrm{Ob_p}}$ and the Wnt–Scl–LRP5/6 signalling pathway through $\pi^{\mathrm{Wnt}}_{\mathrm{act,Ob_p}}$ (see Eq. (43)). As discussed in Buenzli et al. [10], a necessary condition for the $\mathrm{Ob_p}$ population to stay bounded and to converge to a meaningful steady-state (with finite, positive cell densities) is that:

$$\mathcal{P}_{\mathrm{Ob_p}} - \mathcal{D}_{\mathrm{Ob_p}} < 0 \quad \text{as } t \longrightarrow \infty \tag{45}$$

Following Buenzli et al. [10] $P_{\mathrm{Ob_p}}$ was defined considering a saturation factor:

$$P_{\mathrm{Ob_p}} = \begin{cases} P^0_{\mathrm{Ob_p}} \left(1 - \dfrac{\mathrm{Ob_p}}{\mathrm{Ob_p^{sat}}}\right) & \text{if} \quad \mathrm{Ob_p} < \mathrm{Ob_p^{sat}} \\ 0 & \text{if} \quad \mathrm{Ob_p} \geq \mathrm{Ob_p^{sat}} \end{cases} \tag{46}$$

where $P^0_{\mathrm{Ob_p}}$ is a constant and $\mathrm{Ob_p^{sat}}$ is the maximum concentration of osteoblast precursors above which no proliferation occurs. This saturation and the choice of $P^0_{\mathrm{Ob_p}}$ (see Table 2) ensures that Eq. (45) is fulfilled.

9. Adjustment of the constants

In order to adjust all the variables of the model to the data obtained from the literature, it has been necessary to readjust the constants of the model with respect to the one developed by Martin et al. [1]. Next, we will explain how the adjustment has been made and what are the variables to adjust.

The parameters that were readjusted are the ones shown in table 1. Where we have used the value RANKL = 0.5 since in the literature was found to be within the interval 0.3-0.7 (Polyzois et al. [11], Crisafulli et al. [12]). We have considered a $f_{bm} = 43.7\%$, and it was estimated by assuming an average fbm = 93% for cortical bone [13] and fbm = 14% for trabecular one [14].

In this Section we explain how the adjustment of the constants was done for the purpose of matching them with the ones found in the literature. Proceeded as follows:

Variable	Value	Reference
RANKL	0.5 pM	Polyzois et al. [11]
OPG	4 pM	Amrein et al. [15]
NO	1.067e4 pM	Sangwan et al. [16]
Strain level	800-1200 $\mu\varepsilon$	Frost [17]
Bone density loss due to disuse	1-1.5%	LeBlanc et al. [18]

Table 1: Model values that need adjustment taken from the literature

1. Firstly we readjusted the value of the bone density loss due to micro-gravity (disuse), so that we changed the constants A_{OC_a} and γ_{act}
2. Then, the value of OPG was adjusted by changing the constant β_{OPG}.
3. Consecutively, the strain level in homeostasis was readjusted by altering the constant ε_{max0} (See Eq. 47 and Eq. 48:

$$\delta_{rep} = \frac{(\delta_{eq} - \rho_{rep})\,\varepsilon_{max0}}{(\alpha_{rep} - \delta_{eq})^{\frac{1}{\gamma_{rep}}}} \tag{47}$$

$$\delta_{act} = \frac{(\alpha_{act} - \delta_{eq})\,\varepsilon_{max0}}{(\delta_{eq} - \rho_{act})^{\frac{1}{\gamma_{act}}}} \tag{48}$$

   where δ_{eq} is the value of the regulation functions for the homeostatic stress and ε_{max0} is the strain at homeostasis.
4. Fourthly, the value of RANKL was readjusted as follows:
   Considering a fbm = 30%, the parameters that influence the RANKL were modified so that them do not affect everything else. We adjusted RANKL to settled around 1.5 pM at 20 years. For the purpose of keeping constant π_{act,OC_u}^{RANKL}, the value of K_{act,OC_u}^{RANKL} was changed.
   Considering the new value of RANKL, RANK was calculated by substituting the value into the following equation:

$$[RANK] = \frac{N_{OC_p}^{RANK} \cdot OC_p}{1 + \frac{[RANKL]}{K_{RANK-RANKL}}} \tag{49}$$

   where $K_{RANK-RANKL}$ is the dissociation constant of the complex RANK-RANKL and $N_{OC_p}^{RANK}$ is the number of RANK receptors per osteoclast precursor.

With the aim of keeping constant the value of OPG and considering the equation that simulates the evolution of OPG (See Eq. (50)), we have focussed in the fraction shown in Eq. (51).

$$[\mathrm{OPG}] = \frac{P_{\mathrm{OPG}}}{\tilde{D}_{\mathrm{OPG}} + \frac{\tilde{D}_{\mathrm{OPG-RANKL}}\,[\mathrm{RANKL}]}{K_{\mathrm{OPG-RANKL}}}} \tag{50}$$

$$\frac{\tilde{D}_{OPG-RANKL}}{K_{OPG-RANKL}} \cdot RANKL \tag{51}$$

Assuming that the parameter $K_{OPG-RANKL}$ was held constant, $\tilde{D}_{OPG-RANKL}$ was recalculated so that the fraction shown in Eq. 51 would still have the same value.

Next, $[RANKL]_{tot}$ is recalculated as follows:

$$[RANKL]_{tot} = [RANKL] \cdot \left(1 + \frac{[RANK]}{K_{RANK-RANKL}} + \frac{[OPG]}{K_{OPG-RANKL}}\right) \tag{52}$$

In the light of the above, we have the expression of RANKL:

$$[RANKL] = \frac{P_{RANKL}}{\tilde{D}_{RANKL} + \frac{\tilde{D}_{RANK-RANKL}}{K_{RANK-RANKL}} \cdot [RANK] + \frac{\tilde{D}_{OPG-RANKL}}{K_{OPG-RANKL}} \cdot [OPG]} \tag{53}$$

The value of P_{RANKL} is calculated so that the RANKL is settled to the desired value. We have assigned 65% to $P_{OB_p}^{RANKL}$ (See Eq. (54)) and 35% to P_{Ot}^{RANKL} (See Eq. (55)).

$$P_{OB_p}^{RANKL} = \beta_{RANKL,OB_p} \cdot \pi_{act/rep,RANKL}^{PTH,NO} \cdot \left(1 - \frac{[RANKL]_{tot}}{[RANKL]_{max}}\right) \cdot OB_p \tag{54}$$

$$P_{Ot}^{RANKL} = \beta_{RANKL,Ot} \cdot \pi_{act,RANKL}^{dam} \cdot \left(1 - \frac{[RANKL]_{tot}}{[RANKL]_{max}}\right) \cdot Ot \tag{55}$$

From these equations the values of β_{RANKL,OB_p} and $\beta_{RANKL,Ot}$ are obtained.

5. Finally, once RANKL has been readjusted, we adjusted NO by considering that the average bone (20% cortical and 80% trabecular) has a

value of 1.067e4 pM aiming to resemble the literature. Thus, taking the Eq. (32) we have calculated the value of K_{rep}^{NO} in order to keep constant $\pi_{rep,RANKL}^{NO}$ with the new value of NO. Next, the value of $\beta_{NO,Ot}$ has to be calculated for the new NO by using Eq. 33.

If after these modifications the value of the loss of mass due to disuse changes, the whole process would have to be repeated until everything converges.

10. Model constants

The model constants, except for those related to the damage and mineralisation algorithms, which were given in the main document, are provided in the following table.

Constant	Value	Units
Cell constants: differentiation, proliferation, apoptosis, activity		
$\mathrm{Ob_u}$	0.01	pM
$\mathrm{Oc_u}$	0.01	pM
$D_{\mathrm{Ob_u}}$	0.01	day^{-1}
$D_{\mathrm{Ob_p}}$	0.0705	day^{-1}
$P^0_{\mathrm{Ob_p}}$	3.47	day^{-1}
$\mathrm{Ob_p^{sat}}$	0.005	pM
$D_{\mathrm{Oc_u}}$	0.0388	day^{-1}
$D_{\mathrm{Oc_p}}$	0.1216	day^{-1}
$\Delta_{\mathrm{Ob_a}}$	0.3502	day^{-1}
$A_{\mathrm{Oc_a}}$	4.5	day^{-1}
η	$4.143 \cdot 10^{-4}$	pM / %[1]
k_{res}	200	% day^{-1} pM^{-1}
k_{form}	40	% day^{-1} pM^{-1}
RANK-RANKL-OPG signalling pathway		
$\tilde{D}_{\mathrm{OPG}}$	0.35	day^{-1}
$\tilde{D}_{\mathrm{RANKL}}$	0.4053	day^{-1}
$\tilde{D}_{\mathrm{Dmab_{BC}}}$	16.11 *	day^{-1}
$\tilde{D}_{\mathrm{OPG-RANKL}}$	559.67	day^{-1}
$\tilde{D}_{\mathrm{RANK-RANKL}}$	10.132	day^{-1}
$\tilde{D}_{\mathrm{Dmab-RANKL}}$	10.132 *	day^{-1}
$K_{\mathrm{OPG-RANKL}}$	2300	pM
$K_{\mathrm{RANK-RANKL}}$	10	pM

Continued on next page

[1]Recall that f_{bm} is expressed in %.

Constant	Value	Units
$K_{\mathrm{Dmab-RANKL}}$	6.944	pM
$N^{\mathrm{RANK}}_{\mathrm{Oc_p}}$	$4.16 \cdot 10^3$	pM RANK / pM cell
$\beta_{\mathrm{OPG,Ob_a}}$	$1.3 \cdot 10^5$ *	pM OPG / pM cell day^{-1}
$[\mathrm{OPG}]_{\mathrm{max}}$	$1.314 \cdot 10^2$	pM
$[\mathrm{RANKL}]_{\mathrm{max}}$	41.584	pM
$\beta_{\mathrm{RANKL,Ot}}$	$2.836 \cdot 10^3$	pM RANKL / pM cell day^{-1}
$\beta_{\mathrm{RANKL,Ob_p}}$	$2.14 \cdot 10^2$	pM RANKL / pM cell day^{-1}
$K^{\mathrm{RANKL}}_{\mathrm{act,Oc_u}}$	7.487	pM
$K^{\mathrm{RANKL}}_{\mathrm{act,Oc_p}}$	1.497	pM
Upregulation of RANKL via damage		
ρ_{dam}	0.04 *	-
α_{dam}	1 *	-
K_{dam}	0.28 *	-
Competitive binding Wnt-Scl-LRP5/6		
$\tilde{D}^0_{\mathrm{Scl}}$	5	day^{-1}
$\tilde{D}_{\mathrm{Scl-LRP5/6}}$	50	day^{-1}
$K_{\mathrm{Wnt-LRP5/6}}$	$1.079 \cdot 10^3$	pM
$K_{\mathrm{Scl-LRP5/6}}$	8.57	pM
$N^{\mathrm{LRP5/6}}_{\mathrm{OB_p}}$	5	pM LRP5/6 / pM cell
$\beta_{\mathrm{Scl,Ot}}$	$5 \cdot 10^4$ *	pM Scl / pM cell day^{-1}
$[\mathrm{Wnt}]$	170	pM
$[\mathrm{Scl}]_{\mathrm{max}}$	70	pM
$P^0_{\mathrm{Scl,d}}$	0	pM day^{-1}
Co-regulation of RANKL via PTH and NO		
λ_s	0.45	-
λ_c	74.66	-
$K^{\mathrm{PTH}}_{\mathrm{act}}$	0.65	pM
$K^{\mathrm{PTH}}_{\mathrm{rep}}$	0.223	pM
$K^{\mathrm{NO}}_{\mathrm{rep}}$	$5.24 \cdot 10^2$ *	pM
$[\mathrm{NO}]_{\mathrm{max}}$	$2 \cdot 10^8$	pM

Continued on next page

Constant	Value	Units
β_{PTH}	250	pM day $^{-1}$
$\tilde{D}_{\text{PTH}}$	86	day^{-1}
$\beta_{\text{NO,Ot}}$	1.39 $\cdot 10^3$ *	pM NO / pM cell day^{-1}
$\tilde{D}_{\text{NO}}$	2.1 $\cdot 10^{-3}$	day^{-1}
$P_{\text{NO,d}}$	0	pM day^{-1}
	Dmab PK-PD constants *	
k_a	0.17	day^{-1}
k_{el}	$1.15 \cdot 10^{-2}$	day^{-1}
V_c	77.9	ml kg^{-1}
F	1	-
K_m	411	ng ml^{-1}
V_{max}	2672	ng kg^{-1} day^{-1}
ζ	0.65 *	-
	TGF-β related constants	
$\dfrac{\alpha_{\text{TGF}-\beta}\, k_{res}}{\tilde{D}_{\text{TGF}-\beta}}$	1	-
$K_{\text{act}}^{\text{TGF}-\beta}$	$5.633 \cdot 10^{-4}$	pM
$K_{\text{rep}}^{\text{TGF}-\beta}$	$1.754 \cdot 10^{-4}$	pM
	Parameters of mechanical regulation	
α_{rep}	1	-
α_{act}	1	-
ρ_{rep}	0	-
ρ_{act}	0	-
δ_{rep}	2.0 $\cdot 10^{-3}$ *	MPa
δ_{act}	9.37 $\cdot 10^{-4}$ *	MPa
γ_{rep}	6 *	-
γ_{act}	11.9 *	-
δ_{eq}	0.95	-

Table 2: Values taken for the constants of the PK-PD model. All the constants were taken from the model developed by Martin et al. [1], except those marked with an asterisk.

All the constants in Table 2 were taken from the model developed by Martin et al. [1], except those marked with an asterisk. These changes were motivated by the fact that Martin et al. did not considered damage and the variable mineral content of bone matrix. Hence, those constants needed to be readjusted to reproduce the behaviour of the previous model. So, $\beta_{\mathrm{Scl,Ot}}$ and $\beta_{\mathrm{NO,Ot}}$ were readjusted to achieve the same sclerostin and NO levels. The constants of the block "Upregulation of RANKL via damage" are new since damage were not considered in the previous model. Their values were fitted as in [5], so that the contribution of damage to RANKL production is similar in normal situations (homeostasis) to the production of RANKL by osteoblast precursors (see Eq. (11) in the main document). The constants of the block "Dmab PK-PD constants" are specific of the Dmab PK model and are taken from previous works [5, 8]. The degradation rate of the complex RANKL-Dmab, $\tilde{D}_{\mathrm{Dmab-RANKL}}$, was chosen equal to the degradation rates of the other complex involving RANKL, while ζ and $\tilde{D}_{\mathrm{Dmab_{BC}}}$ were adjusted as explained in section 3.1 of the main document. The constants of the block "PMO related constants" were readjusted as done in [1], i.e. to reproduce the results of the longitudinal experimental study of Nordin et al. [19] on the evolution of the bone mineral density (BMD) in the forearm of post-menopausal women. Martin et al. [1] did not account for variations in mineral content and assumed the bone matrix fraction to evolve in the same way as the BMD. Now that this limitation has been overcome by including the mineral content, it was necessary to readjust the constants.

References

[1] M. Martin, V. Sansalone, D.M.L. Cooper, M.R. Forwood, P. Pivonka. Mechanobiological osteocyte feedback drives mechanostat regulation of bone in a multiscale computational model. Biomech Model Mechan 2019;18(5):1475–96. doi:10.1007/s10237-019-01158-w.

[2] P. Pivonka, J. Zimak, D.W. Smith, et al. Model structure and control of bone remodeling: A theoretical study. Bone 2008;43(2):249–63. doi:10.1016/j.bone.2008.03.025.

[3] P. Pivonka, P. Buenzli, C.R. Dunstan. A Systems Approach to Understanding Bone Cell Interactions in Health and Disease. USA: InTech. ISBN 9789535107927; 2012, p. 169–204. doi:10.5772/51149.

[4] M.C. Peterson, M.M. Riggs. A physiologically based mathematical model of integrated calcium homeostasis and bone remodeling. Bone 2010;46(1):49–63. doi:10.1016/j.bone.2009.08.053.

[5] J. Martínez-Reina, J.L. Calvo-Gallego, P. Pivonka. Combined effects of exercise and denosumab treatment on local failure in post-menopausal osteoporosis — insights from bone remodelling simulations accounting for mineralisation and damage. Front Bioeng Biotechnol 2021;9:635056. doi:10.3389/fbioe.2021.635056.

[6] J. Martínez-Reina, J. L. Calvo-Gallego, M. Martin, P. Pivonka. Assessment of strategies for safe drug discontinuation and transition of denosumab treatment in pmo—insights from a mechanistic pk/pd model of bone turnover. Front Bioeng Biotechnol 2022;10:886579. doi:10.3389/fbioe.2022.886579.

[7] J. Martínez-Reina, P. Pivonka. Effects of long-term treatment of denosumab on bone mineral density: insights from an in-silico model of bone mineralization. Bone 2019;125:87–95. doi:10.1016/j.bone.2019.04.022.

[8] J. Martínez-Reina, J. L. Calvo-Gallego, P. Pivonka. Are drug holidays a safe option in treatment of osteoporosis? – Insights from an in silico mechanistic PK–PD model of denosumab treatment of postmenopausal osteoporosis. J Mech Behav Biomed 2021;113:104140. doi:10.1016/j.jmbbm.2020.104140.

[9] D. D. Marathe, A. Marathe, D. E. Mager. Integrated model for denosumab and ibandronate pharmacodynamics in postmenopausal women. Biopharm Drug Dispos 2011;32(8):471–81. doi:10.1002/bdd.770.

[10] P. R. Buenzli, P. Pivonka, B. S. Gardiner, D. W. Smith. Modelling the anabolic response of bone using a cell population model. J Theor Biol 2012;307:42–52. doi:10.1016/j.jtbi.2012.04.019.

[11] M. Polyzois, S. A. Polyzos, A. D. Anastasilakis, E. Terpos, G. Kanakis, et al. Serum osteoprotegerin, RANKL, and Dkk-1 levels in adults with Langerhans cell histiocytosis. J Clin Endocr Metab 2012;97(4):E618–21. doi:10.1210/jc.2011-2962.

[12] A. Crisafulli, A. Micari, D. Altavilla, F. Saporito, A. Sardella, et al. Serum levels of osteoprotegerin and RANKL in patients with ST elevation acute myocardial infarction. Clin Sci (Lond) 2005;109(4):389–95. doi:10.1042/CS20050058.

[13] L. Cardoso, S. P. Fritton, G. Gailani, M. Benalla, S. C. Cowin. Advances in assessment of bone porosity, permeability and interstitial fluid flow. J Biomech 2013;46(2):253–65. doi:10.1016/j.jbiomech.2012.10.025.

[14] D. Ulrich, B. van Rietbergen, A. Laib, P. Rüegsegger. The ability of three-dimensional structural indices to reflect mechanical aspects of trabecular bone. Bone 1999;25(1):55–60. doi:10.1016/S8756-3282(99)00098-8.

[15] K. Amrein, S. Amrein, C. Drexler, H. P. Dimai, H. Dobnig, et al. Sclerostin and its association with physical activity, age, gender, body composition, and bone mineral content in healthy adults. J Clin Endocrinol Metab 2012;97(1):148–54. doi:10.1210/jc.2011-2152.

[16] L. Sangwan, R. Kumar, R. Peter, P. Arun. Evaluation of nitric oxide levels in chronic myeloid leukemia. Int J Innov Res Rev 2014;2(2):1–5.

[17] H. M. Frost. Defining osteopenias and osteoporoses: another view (with insights from a new paradigm). Bone 1997;20(5):385–91. doi:10.1016/S8756-3282(97)00019-7.

[18] A. D. LeBlanc, E. R. Spector, H. J. Evans, J. D. Sibonga. Skeletal responses to space flight and the bed rest analog: a review. J Musculoskel Neuron 2007;7(1):33–47.

[19] B. E. Nordin, A. G. Need, B. E. Chatterton, M. Horowitz, H. A. Morris. The relative contributions of age and years since menopause to postmenopausal bone loss. J Clin Endocrinol Metab 1990;70(1):83–8. doi:10.1210/jcem-70-1-83.
